# Identifying key brain pathology in bipolar and unipolar depression using a region-specific brain aging trajectories approach: Insights from the Taiwan Aging and Mental Illness Cohort

**DOI:** 10.1017/S0033291725101517

**Published:** 2025-08-29

**Authors:** Jun-Ding Zhu, I-Jou Chi, Hui-Yun Hsu, Shih-Jen Tsai, Albert C. Yang

**Affiliations:** 1 Department of Occupational Therapy, College of Medical Science and Technology, Chung Shan Medical University, Taichung, Taiwan; 2 Occupational Therapy Room, Chung Shan Medical University Hospital, Taichung, Taiwan; 3 Institute of Brain Science, National Yang Ming Chiao Tung University, Taipei, Taiwan; 4 Digital Medicine and Smart Healthcare Research Center, National Yang Ming Chiao Tung University, Taipei, Taiwan; 5 Department of Psychiatry, Taipei Veteran General Hospital, Taipei, Taiwan; 6 School of Medicine, National Yang Ming Chiao Tung University, Taipei, Taiwan; 7 Department of Medical Research, Taipei Veteran General Hospital, Taipei, Taiwan

**Keywords:** brain aging trajectories, bipolar disorder, major depressive disorder, neuroimaging, region-specific model, machine learning, gray matter, standard deviation, fractional anisotropy, magnetic resonance imaging, diffusion tensor imaging, mental illness

## Abstract

**Background:**

Identifying key areas of brain dysfunction in mental illness is critical for developing precision diagnosis and treatment. This study aimed to develop region-specific brain aging trajectory prediction models using multimodal magnetic resonance imaging (MRI) to identify similarities and differences in abnormal aging between bipolar disorder (BD) and major depressive disorder (MDD) and pinpoint key brain regions of structural and functional change specific to each disorder.

**Methods:**

Neuroimaging data from 340 healthy controls, 110 BD participants, and 68 MDD participants were included from the Taiwan Aging and Mental Illness cohort. We constructed 228 models using T1-weighted MRI, resting-state functional MRI, and diffusion tensor imaging data. Gaussian process regression was used to train models for estimating brain aging trajectories using structural and functional maps across various brain regions.

**Results:**

Our models demonstrated robust performance, revealing accelerated aging in 66 gray matter regions in BD and 67 in MDD, with 13 regions common to both disorders. The BD group showed accelerated aging in 17 regions on functional maps, whereas no such regions were found in MDD. Fractional anisotropy analysis identified 43 aging white matter tracts in BD and 39 in MDD, with 16 tracts common to both disorders. Importantly, there were also unique brain regions with accelerated aging specific to each disorder.

**Conclusions:**

These findings highlight the potential of brain aging trajectories as biomarkers for BD and MDD, offering insights into distinct and overlapping neuroanatomical changes. Incorporating region-specific changes in brain structure and function over time could enhance the understanding and treatment of mental illness.

## Introduction

Identifying key areas of brain dysfunction in mental illness is critical for developing precision diagnosis and treatment in psychiatry (Zhang, Braun, Tost, & Bassett, 2020). Despite numerous efforts, the neuropathological mechanisms behind mental illness remain elusive. From the clinical perspective, observing and quantifying brain abnormalities may help develop new, noninvasive treatment strategies, such as personalized repetitive transcranial magnetic stimulation (Gogulski et al., 2023) or noninvasive deep brain stimulation (Vassiliadis et al., 2024; Violante et al., 2023). The challenge lies in reliably identifying crucial brain regions as the target for treatment. Traditional cross-sectional approaches comparing healthy participants with patients are limited (Cornblath, Lydon-Staley, & Bassett, 2019), as they do not consider changes in brain structure and function over time in healthy aging or how these trends may be altered in mental illness through progressive illness (Shen, Tsai, Lin, & Yang, 2023). Therefore, statistical or machine learning approaches that classify mental illness versus healthy states may be less helpful due to their inability to account for dynamic changes in mental illness (Cornblath et al., 2019).

Introducing time variables, such as biological age or the duration of illness, allows for observing dynamic changes in brain structure and function throughout the illness. We propose that identifying key brain abnormalities in mental illness might be facilitated by a region-specific brain age prediction approach (Zhu, Tsai, Lin, Lee, & Yang, 2023; Zhu, Wu, Tsai, Lin, & Yang, 2023). Brain age prediction uses machine learning to model the trajectory of changes in brain structure and function across the lifespan in a healthy cohort and employs this model to predict deviations of brain age (or brain age gap, [BAG]) in neuropsychiatric disorders. However, traditional brain age prediction considers only whole-brain features and provides less insight into regional changes in structure and function (Franke & Gaser, 2019; Franke, Luders, May, Wilke, & Gaser, 2012). Previously, we have developed a region-specific approach to estimate the trajectory of brain structure and function in individual brain regions, thereby identifying key brain abnormalities in schizophrenia throughout the course of the illness (Zhu, Tsai, et al., 2023; Zhu, Wu, et al., 2023).

Bipolar disorder (BD) and major depressive disorder (MDD) are affective disorders also known to exhibit structural and functional brain changes over time. Studies indicated that patients with BD experienced significant reductions in gray matter density in regions such as the hippocampus and cerebellum, linked to cognitive deterioration and mood episodes (Moorhead et al., 2007). However, meta-analyses on BD and brain aging have yielded inconsistent results (Ballester et al., 2021). MDD is associated with structural abnormalities in brain regions such as the hippocampus and frontal cortex, which can serve as predictive biomarkers for treatment response (Kang & Cho, 2020). Yet, evidence on accelerated brain aging in MDD is also inconsistent (Ballester et al., 2022; Bashyam et al., 2020; Christman et al., 2020). Previous studies found significant brain age differences in MDD using structural covariance networks and functional magnetic resonance imaging (fMRI), linking older brain age to increased impulsivity and depression severity (Dunlop, Victoria, Downar, Gunning, & Liston, 2021; Kuo et al., 2020). Despite these findings, no current study has applied a region-specific brain age prediction approach to examine regional changes in brain structure and function in affective disorders throughout the course of the illness. Such an approach may provide deeper insights into their neuropathology and the similarities or distinctions between BD and MDD.

We hypothesized that affective disorders cause structural and functional brain abnormalities, altering brain aging trajectories. To understand their impact, region-specific brain age prediction models were constructed using multiple neuroimaging modalities. Therefore, this study aimed to (1) develop multimodal region-specific brain age prediction models for different brain regions using T1-weighted MRI, resting-state fMRI, and diffusion tensor imaging (DTI), (2) identify similarities and differences in abnormal aging between BD and MDD across different brain regions, and (3) identify key brain regions of structural and functional change specifically for BD or MDD.

## Methods

### Participants

Participants in this study were sourced from the Taiwan Aging and Mental Illness (TAMI) cohort (Shen et al., 2023; Yang et al., 2015; Yang, Tsai, Lin, Peng, & Huang, 2018; Zhu, Tsai, et al., 2023; Zhu, Wu, et al., 2023). We included 230 healthy controls (HCs) aged 20 to 84 for the training dataset and validated the results with an independent test dataset of 110 HCs (see Supplementary Methods and Supplementary Figure S1 for further details). This study also included 110 individuals with BD (i.e., bipolar I disorder) as well as 68 individuals with MDD (i.e., unipolar depression) from the TAMI cohort. The diagnosis of mental illness was based on the Diagnostic and Statistical Manual of Mental Disorders, Fourth Edition, Text Revision. To ensure diagnostic reliability, all diagnoses were further confirmed using the Mini-International Neuropsychiatric Interview, a structured diagnostic interview (Sheehan et al., 1998). Detailed exclusion criteria and clinical assessments are provided in the Supplementary Material. Two control groups of equal size were randomly selected from the independent test dataset for subsequent comparison based on the sample size and sex ratio of the BD and MDD groups. Demographic and clinical characteristics of participants are shown in Table 1. The study was conducted in accordance with the Declaration of Helsinki, and the protocol was approved by the institutional review board of Taipei Veterans General Hospital, Taiwan (2023-12-003A). Obtaining informed consent was exempted by the Institutional Review Board because the data were deidentified in the TAMI cohort.

**Table 1.** Demographic and clinical characteristics of the BD and MDD groups, along with sex- and age-matched healthy controls

Characteristics	BD group (n = 110)	HC group (n = 110)	Statistic (t or 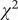 )	*P* value	MDD group (n = 68)	HC group (n = 68)	Statistic (t or 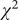 )	*P* value
Age, year	49.35 ± 12.77	49.13 ± 12.68	0.13	0.90^a^	49.04 ± 10.36	48.97 ± 10.06	0.04	0.97^a^
Sex								
Male, n (%)	38 (34.5%)	38 (34.5%)	0.00	1.00^b^	22 (32.4%)	22 (32.4%)	0.00	1.00^b^
Female, n (%)	72 (65.5%)	72 (65.5%)			46 (67.6%)	46 (67.6%)		
Years of education	12.65 ± 3.66	15.51 ± 4.20	−5.40	<0.001^a^	12.51 ± 3.71	15.38 ± 4.37	−4.11	<0.001^a^
MMSE	26.94 ± 3.35	28.75 ± 1.25	−5.30	<0.001^a^	26.96 ± 3.05	28.65 ± 1.28	−4.21	<0.001^a^
Duration of illness, year	20.91 ± 12.80^c^				7.26 ± 8.35^g^			
YMRS	3.29 ± 4.36^d^	–	–	–	–	–	–	–
HAM-D–21	5.49 ± 5.20^e^	–	–	–	10.75 ± 7.06^h^	–	–	–
HAM-A	4.96 ± 4.99^f^	–	–	–	8.64 ± 6.18^i^	–	–	–

*Note*: BD, bipolar disorder; HAM-A, Hamilton Anxiety Rating Scale; HAM-D-21, Hamilton Depression Rating Scale; HC, healthy control; MDD, major depressive disorder; MMSE, Mini-Mental State Examination; TAMI, Taiwan Aging and Mental Illness; YMRS, Young Mania Rating Scale.

a Independent *t* test, significance level = 0.05.

b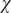 *^2^* test, significance level = 0.05.

c Duration of illness data were available for only 92 participants with BD in the TAMI cohort.

d Only 102 participants with BD had YMRS scores in the TAMI cohort.

e Only 108 participants with BD had HAM-D-21 scores in the TAMI cohort.

f Only 97 participants with BD had HAM-A scores in the TAMI cohort.

g Duration of illness data were available for only 38 participants with MDD in the TAMI cohort.

h Only 67 participants with MDD had HAM-D-21 scores in the TAMI cohort.

i Only 64 participants with MDD had HAM-A scores in the TAMI cohort.

### Image acquisition and preprocessing

**Figure 1.**
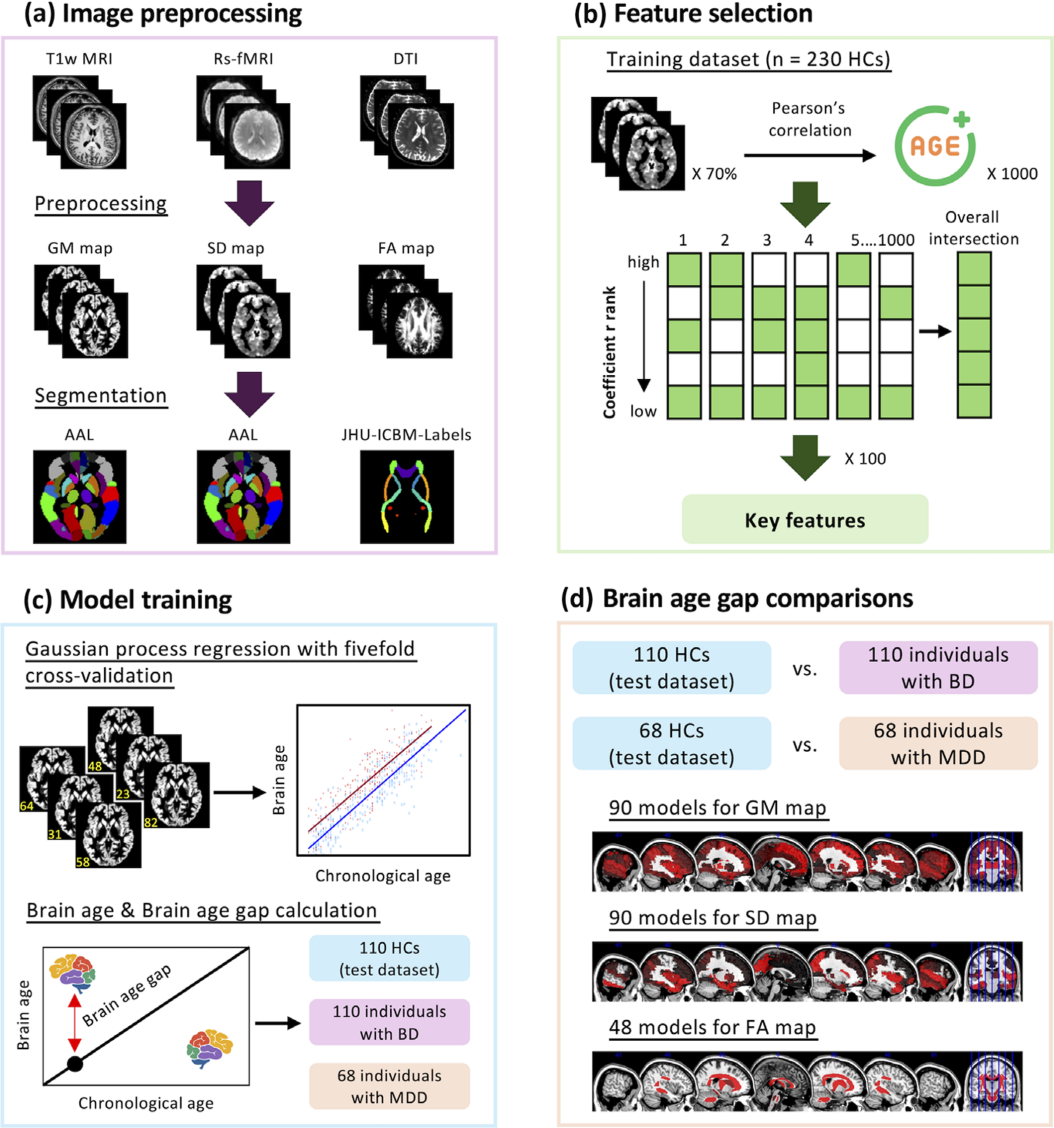
Flow of data preprocessing and construction of the brain aging trajectory models. (a) Subplot a illustrates the neuroimaging preprocessing pipeline used for T1-weighted MRI, resting-state fMRI, and DTI. Images were preprocessed using DPABI, SPM12, and FSL. AAL and JHU-ICBM-Labels-1 mm atlases were applied for image segmentation, resulting in 90 gray matter, 90 standard deviation, and 48 fractional anisotropy maps for further analysis. (b) Pearson’s correlation coefficient was calculated between voxels and chronological age for 70% of the participants randomly selected from the training dataset. To ensure robustness, we repeated this process 1,000 times. We identified key voxels by selecting the top 50% of voxels with the highest correlation coefficient and made intersections across these iterations. We performed this identification process 100 times to generate 100 sets of key voxels. Each voxel selected from these 100 sets was then used as a key feature for model training. (c) The Gaussian process regression algorithm with fivefold cross-validation was utilized to train 228 brain age prediction models. Model performance was evaluated by calculating MAEs and Pearson’s correlation coefficient between corrected brain age and chronological age. The trained models were subsequently applied to the test dataset (*n* = 110) as well as datasets from individuals with BD (*n* = 110) and MDD (*n* = 68) to predict brain age and calculate BAG. (d) Finally, we conducted an ANCOVA to test the BAG differences between individuals with BD and MDD compared to age- and sex-matched healthy controls across different brain regions. *Note*: AAL, automated anatomical labeling; BD, bipolar disorder; BAG, brain age gap; DTI, diffusion tensor imaging; DPABI, Data Processing & Analysis for Brain Imaging; FA, fractional anisotropy; FSL, FMRIB Software Library; GM, gray matter; HC, healthy control; MAE, mean absolute error; MDD, major depressive disorder; rs-fMRI, resting-state functional MRI; SD, standard deviation; SPM, Statistical Parametric Mapping; T1w MRI, T1-weighted MRI.

The MRI data of the participants were acquired using a 3T MRI scanner (Siemens Magnetom Tim Trio, Erlangen, Germany) with a 12-channel head coil at National Yang Ming Chiao Tung University. The scanning protocols were consistent with those used in our previous studies (Shen et al., 2023; Zhu, Tsai, et al., 2023; Zhu, Wu, et al., 2023). The Supplementary Material provides additional information on the scanning protocols used for T1-weighted MRI, resting-state fMRI, and DTI. Briefly, Statistical Parametric Mapping 12 and the Data Processing & Analysis for Brain Imaging toolbox (Yan, Wang, Zuo, & Zang, 2016) were used to preprocess the raw T1-weighted MRI and raw resting-state fMRI data for each participant, operating within MATLAB R2022a (MathWorks, Natick, MA, USA). The DTI data were preprocessed using the FMRIB Software Library version 6.0 (Jenkinson, Beckmann, Behrens, Woolrich, & Smith, 2012). More information can be found in Figure 1a and the Supplementary Material.

### Structural and functional brain maps for region-specific brain age trajectory models

This study used a systematic approach to identify key features represented by voxels that exhibit the strongest correlations with chronological age across different brain regions (Figure 1b). We chose to use gray matter intensity, the standard deviation of resting-state fMRI signals, and fractional anisotropy at the voxel level as features to represent the structural and functional maps of the brain. In particular, the standard deviation of resting-state fMRI signals represented the variability of the hemodynamic response to neuronal activity, which can be an intuitive indicator of brain activation. There is evidence that alterations in the variability of brain signals have been observed in healthy aging (Xie et al., 2020) and patients with mental illness (Li et al., 2019; Sheng et al., 2021; Xie et al., 2018).

Next, we applied the Automated Anatomical Labeling (Tzourio-Mazoyer et al., 2002) and JHU-ICBM-Labels-1 mm atlas (Hua et al., 2008; Wakana et al., 2007) to parcellate gray matter and white matter into 90 regions and 48 tracts, respectively. Then, we constructed corresponding brain aging trajectory models using voxels within a given brain region as features. In total, we constructed 228 brain aging trajectory models, including 90 for gray matter, 90 for standard deviation, and 48 for fractional anisotropy maps. The detailed steps of feature selection are described in our previous studies (Zhu et al., 2022; Zhu, Wu, et al., 2023) and are provided in the Supplementary Material.

### Brain aging trajectory models

We used a Gaussian process regression (GPR) algorithm to train 228 models for estimating brain aging trajectories. GPR is known for its effectiveness in handling complex datasets and has been successful in previous studies (Hope, Seghier, Leff, & Price, 2013; Macke, Gerwinn, White, Kaschube, & Bethge, 2011; Wassermann, Bloy, Kanterakis, Verma, & Deriche, 2010; Ziegler, Ridgway, Dahnke, Gaser, & Initiative, 2014). The models were constructed using voxel features within 90 gray matter, 90 standard deviation, and 48 fractional anisotropy maps from the training dataset. To ensure the models’ generalizability, a fivefold cross-validation method was implemented. The models were also tested on an independent test dataset to evaluate their reproducibility and stability and to predict the brain aging trajectory of all participants. A brain age correction procedure was used to correct potential bias in predicting brain aging trajectory, such as underestimation in older participants and overestimation in younger participants (de Lange & Cole, 2020). We applied the brain age correction procedure as outlined in a previous study (Beck et al., 2022; de Lange & Cole, 2020). First, we established a linear model to describe the relationship between brain age and chronological age using the following equation:

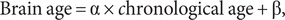

where α represents the slope and β represents the intercept. Given that initial brain age estimates can exhibit systematic bias, we applied a correction to improve its accuracy:

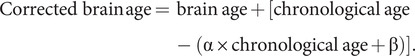

Furthermore, the BAG for a given brain region was calculated as the difference between the corrected region-specific brain age and the chronological age (Figure 1c).

### Statistical analysis

The performances of region-specific brain aging trajectory models were evaluated by calculating the mean absolute error (MAE) and Pearson’s correlation coefficient between the corrected region-specific brain age and the chronological age.

Analysis of covariance (ANCOVA) was employed to test the BAG differences between the BD and control groups, as well as between the MDD and control groups, using chronological age, sex, mini-mental state examination (MMSE) scores, and years of education as covariates to control for potential confounding variables (Figure 1d). In this study, the brain age correction procedure incorporated chronological age into the formula, further reducing variance and MAE while strengthening the correlation between corrected brain age and chronological age. Based on previous research (de Lange & Cole, 2020; Le et al., 2018), we conducted ANCOVA analyses with chronological age as a covariate to examine differences in BAG, aiming to minimize potential age dependence in our findings. The false discovery rate (FDR) method was applied to address multiple comparisons (Benjamini & Yekutieli, 2001), with an adjusted *P* value threshold set at 0.05. Additionally, partial η^2^ values were calculated to measure the effect size, providing insight into the magnitude of the observed differences in the gray matter volume, standard deviation of resting-state fMRI, and fractional anisotropy of white matter tracts between patients with affective disorders and control subjects. All analyses were performed using MATLAB 2023b.

Partial correlation analysis was performed on brain regions exhibiting accelerated aging to examine the association between the BAG and clinical factors (i.e. the Young Mania Rating Scale for the BD group and the Hamilton Depression Rating Scale, Hamilton Anxiety Rating Scale, MMSE, and durations of illness for both the BD and MDD groups) while controlling for chronological age and sex.

## Results

### Performance of brain aging trajectory models

The performance of models for gray matter, standard deviation, and fractional anisotropy maps demonstrated consistent MAEs and robust correlations before and after bias correction across different gray matter regions and white matter tracts. Detailed results for the performance of each model can be found in the Supplementary Material (Supplementary Results and Supplementary Tables S1 to S3).

### BAG differences in gray matter maps between BD/MDD and HC groups

**Figure 2.**
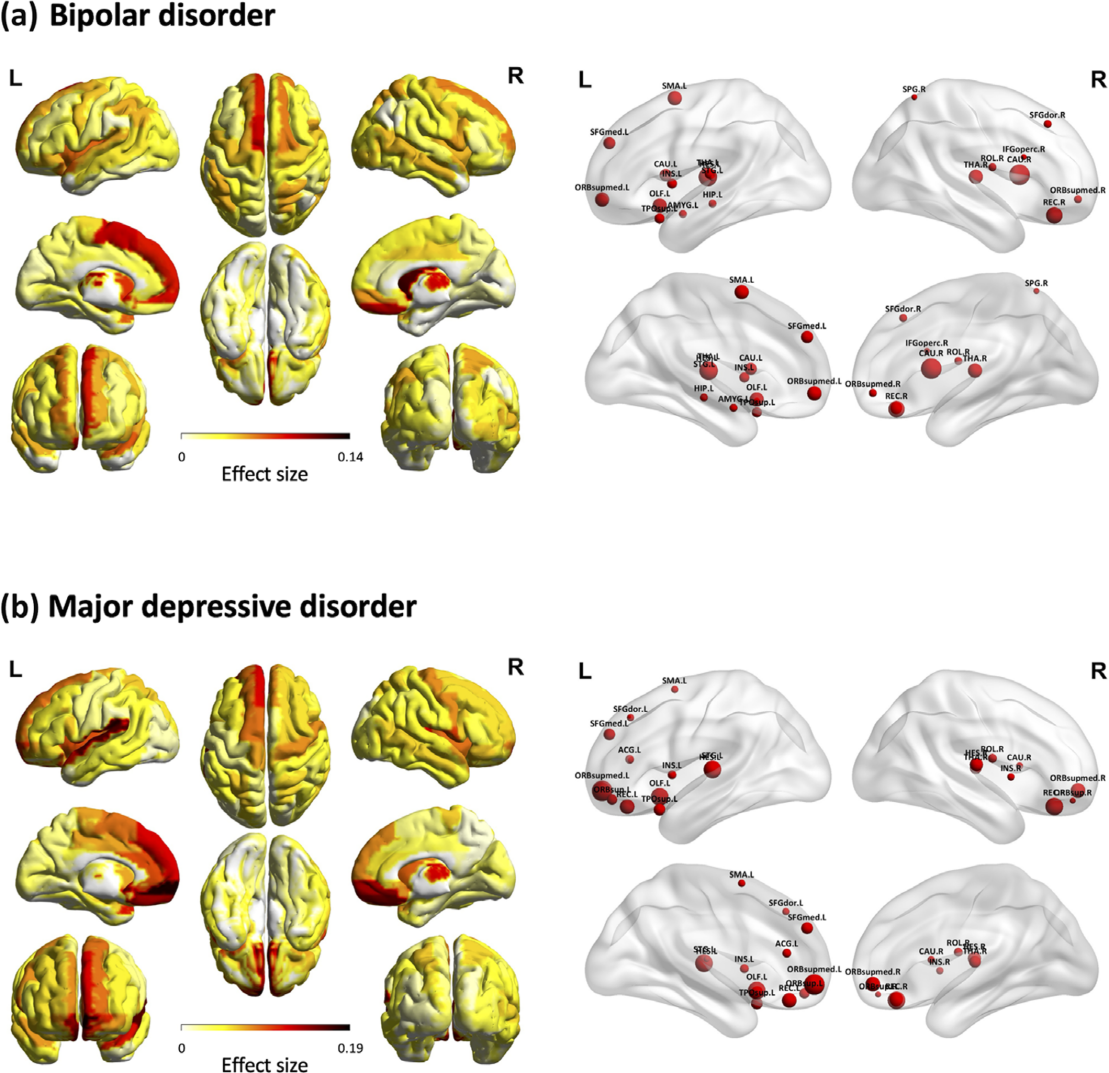
BAG differences between individuals with BD and MDD compared to age- and sex-matched healthy controls in 90 models for gray matter map. (a) The left panel displays the brain regions that showed significantly accelerated aging in individuals with BD, along with their effect sizes, following FDR correction. The color bar represents the effect size (*partial η^2^*). The right panel presents the 20 brain regions with the most significant accelerated aging. The size of each red sphere represents the effect size, with larger spheres indicating greater effect sizes. (b) Similarly, after applying FDR correction, the left panel displays the brain regions that exhibited significantly accelerated aging in individuals with MDD and their effect sizes. The 20 brain regions with the most significant accelerated aging are presented on the right panel. *Note*: ACG.L, left anterior cingulate and paracingulate gyri; AMYG.L, left amygdala; BD, bipolar disorder; BAG, brain age gap; CAU.L, left caudate nucleus; CAU.R, right caudate nucleus; FDR, false discovery rate; HES.L, left Heschl’s gyrus; HES.R, right Heschl’s gyrus; HIP.L, left hippocampus; IFGoperc.R, right inferior frontal gyrus (opercular part); INS.L, left insula; INS.R, right insula; MDD, major depressive disorder; OLF.L, left olfactory cortex; ORBsup.L, left superior frontal gyrus (orbital part); ORBsup.R, right superior frontal gyrus (orbital part); ORBsupmed.L, left superior frontal gyrus (medial orbital); ORBsupmed.R, right superior frontal gyrus (medial orbital); REC.L, left gyrus rectus; REC.R, right gyrus rectus; ROL.R, right rolandic operculum; SFGdor.L, left superior frontal gyrus (dorsolateral); SFGdor.R, right Superior frontal gyrus (dorsolateral); SFGmed.L, left Superior frontal gyrus (medial); SMA.L, left supplementary motor area; SPG.R, right superior parietal gyrus; STG.L, left superior temporal gyrus; THA.L, left thalamus; THA.R, right thalamus; TPOsup.L, left temporal pole (superior temporal gyrus).

For the 90 models of the gray matter maps, 66 brain regions showed significant accelerated aging (i.e., reduction in gray matter volume) in the BD group (Figure 2a). The top 20 regions that exhibited the most abnormal acceleration in aging included the bilateral superior frontal gyrus (dorsolateral), right inferior frontal gyrus (opercular part), right rolandic operculum, left supplementary motor area, left olfactory cortex, left superior frontal gyrus (medial), bilateral superior frontal gyrus (medial orbital), right gyrus rectus, left insula, left hippocampus, left amygdala, right superior parietal gyrus, bilateral caudate nucleus, bilateral thalamus, left Heschl’s gyrus, and the left temporal pole (superior temporal gyrus). Detailed results are listed in Supplementary Table S4.

Significant accelerated aging in gray matter maps was observed in 67 brain regions for the MDD group (Figure 2b). The top 20 regions that showed the most pronounced acceleration in aging within the MDD group included the left superior frontal gyrus (dorsolateral), bilateral superior frontal gyrus (orbital part), right rolandic operculum, left supplementary motor area, left olfactory cortex, left superior frontal gyrus (medial), bilateral superior frontal gyrus (medial orbital), bilateral gyrus rectus, bilateral insula, left anterior cingulate and paracingulate gyri, right caudate nucleus, right thalamus, bilateral Heschl’s gyrus, left superior temporal gyrus, and the left temporal pole (superior temporal gyrus). Detailed results are listed in Supplementary Table S4.

### BAG differences in the standard deviation maps between BD/MDD and HC groups

**Figure 3.**
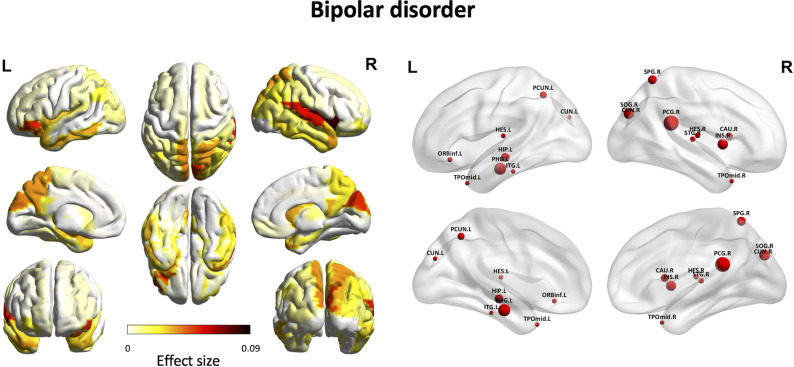
BAG differences between individuals with BD and age- and sex-matched healthy controls in 90 models for the standard deviation map. The left panel displays the brain regions that showed significantly accelerated aging in individuals with BD, along with their effect sizes, following FDR correction. The color bar represents the effect size (*partial η^2^*). The right panel presents the 17 brain regions with significant accelerated aging. The size of each red sphere represents the effect size, with larger spheres indicating greater effect sizes. *Note*: BD, bipolar disorder; BAG, brain age gap; CAU.R, right caudate nucleus; CUN.L, left cuneus; CUN.R, right cuneus; FDR, false discovery rate; HES.L, left Heschl’s gyrus; HES.R, right Heschl’s gyrus; HIP.L, left hippocampus; INS.R, right insula; ITG.L, left inferior temporal gyrus; ORBinf.L, left inferior frontal gyrus (orbital part); PCG.R, right posterior cingulate gyrus; PCUN.L, left precuneus; PHG.L, left parahippocampal gyrus; SOG.R, right superior occipital gyrus; SPG.R, right superior parietal gyrus; STG.R, right superior temporal gyrus; TPOmid.L, left temporal pole (middle temporal gyrus); TPOmid.R, right temporal pole (middle temporal gyrus).

Analysis of BAG based on the standard deviation maps showed that 17 brain regions in the BD group exhibited accelerated aging (i.e. reduced standard deviation of resting-state fMRI) out of the 90 regions examined (Figure 3). These brain regions included the left inferior frontal gyrus (orbital part), the right insula, the right posterior cingulate gyrus, the left hippocampus, the left parahippocampal gyrus, the bilateral cuneus, the right superior occipital gyrus, the right superior parietal gyrus, the left precuneus, the right caudate nucleus, the bilateral Heschl’s gyrus, the right superior temporal gyrus, the bilateral temporal pole (middle temporal gyrus), and the left inferior temporal gyrus. Detailed results are listed in Supplementary Table S5. Conversely, the analysis did not show any statistically significant accelerated brain aging in the MDD group after applying the FDR correction (Supplementary Table S5).

### BAG differences in fractional anisotropy maps between BD/MDD and HC groups

**Figure 4.**
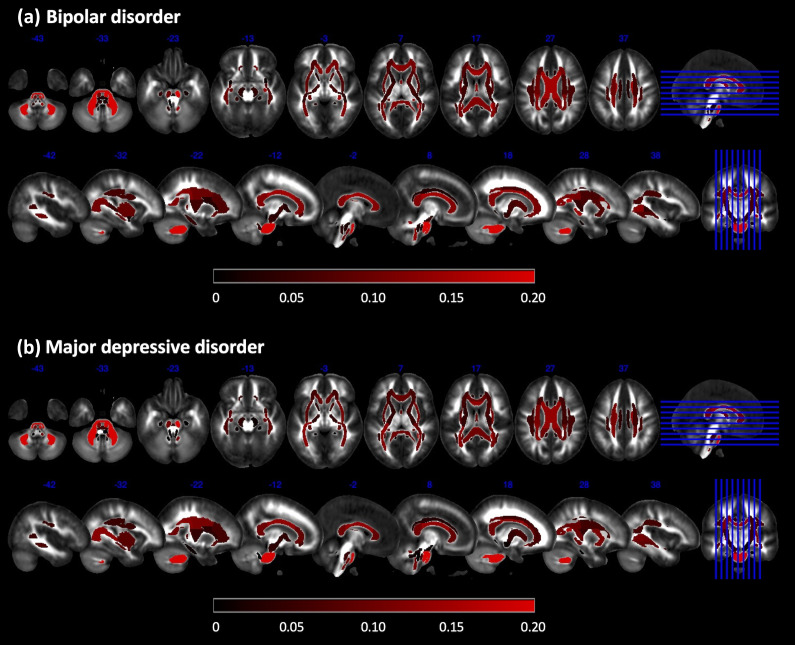
BAG differences between individuals with BD and MDD compared to age- and sex-matched healthy controls in 48 models for the fractional anisotropy map. (a) The white matter tracts that exhibited significantly accelerated aging in individuals with BD, along with their effect sizes, following FDR correction. The color bar represents the effect size (*partial η^2^*). (b) Similarly, the white matter tracts showed significantly accelerated aging in individuals with MDD, along with their effect sizes, after applying FDR correction. *Note*: BD, bipolar disorder; BAG, brain age gap; FDR, false discovery rate; MDD, major depressive disorder.

Of the 48 white matter tracts analyzed, 43 exhibited accelerated aging (i.e. reduced fractional anisotropy) in individuals with BD (Figure 4a). The top 20 white matter tracts showing the highest degree of accelerated aging included the middle cerebellar peduncle, corpus callosum, fornix (column and body), bilateral corticospinal tracts, right inferior cerebellar peduncle, right anterior corona radiata, left superior corona radiata, bilateral posterior corona radiata, bilateral posterior thalamic radiation, left sagittal stratum, left external capsule, bilateral fornix (cres)/stria terminalis, and bilateral tapetum. Detailed results are listed in Supplementary Table S6.

In the MDD group, 39 of 48 white matter tracts exhibited accelerated aging (Figure 4b). The top 20 most significantly aged tracts included the middle cerebellar peduncle, corpus callosum, fornix (column and body), bilateral corticospinal tracts, right inferior cerebellar peduncle, bilateral retrolenticular part of the internal capsule, left superior corona radiata, bilateral posterior corona radiata, bilateral posterior thalamic radiation, right sagittal stratum, right external capsule, right fornix (cres)/stria terminalis, and bilateral tapetum. Detailed results are listed in Supplementary Table S6.

### Common and distinct brain regions with significant BAG differences between BD/MDD and HC groups

For the gray matter maps, 13 of the 20 brain regions showing the most significant deterioration were common to both the BD and MDD groups. The affected brain regions included the left superior frontal gyrus (dorsolateral), right rolandic operculum, left supplementary motor area, left olfactory cortex, left superior frontal gyrus (medial), bilateral superior frontal gyrus (medial orbital), right gyrus rectus, left insula, right caudate nucleus, right thalamus, left Heschl’s gyrus, and the left temporal pole (superior temporal gyrus).

The BD group demonstrated significant aging in unique brain regions, including the right inferior frontal gyrus (opercular part), left hippocampus, left amygdala, and right superior parietal gyrus. In contrast, unique structural abnormalities in MDD were localized to specific brain regions, namely the left anterior cingulate and paracingulate gyri and the bilateral superior frontal gyrus (orbital part).

For functional brain maps, there were no significantly accelerated aging regions in the MDD group, so no common or unique brain regions were reported for the BD and MDD groups.

For fractional anisotropy maps, 16 tracts were common between the BD and MDD groups. The shared white matter tracts included the middle cerebellar peduncle, the genu of the corpus callosum, the body of the corpus callosum, the splenium of the corpus callosum, the fornix (column and body), bilateral corticospinal tracts, the right inferior cerebellar peduncle, the left superior corona radiata, bilateral posterior corona radiata, bilateral posterior thalamic radiation, the right fornix (cres)/stria terminalis, and bilateral tapetum.

Additionally, illness-specific accelerated aging in the BD group was found within the right anterior corona radiata, while the MDD group showed a unique characteristic of the bilateral retrolenticular part of the internal capsule.

### Correlations of BAG with clinical assessments and durations of illness

Our results showed that, after applying FDR correction, no statistically significant correlations were found between the BAG and any clinical assessment scores or duration of illness in either group, except for a significant negative correlation between BAG derived from the fractional anisotropy map in the left anterior limb of the internal capsule and MMSE scores in the BD group (*r* = −0.345, adjusted *P* = 0.011).

## Discussion

This study revealed four main findings: first, the region-specific brain aging trajectory models demonstrated consistent MAEs and robust correlations between corrected brain age and chronological age across various gray matter regions and white matter tracts. Second, FDR-adjusted comparisons highlighted those 66 gray matter regions in the BD group and 67 regions in the MDD group exhibited accelerated aging, with some overlap and unique regions for each disorder. Third, analysis of standard deviation maps revealed accelerated aging in 17 brain regions for the BD group but no significant regions for the MDD group. Finally, analysis of fractional anisotropy indicated that 43 white matter tracts in BD and 39 in MDD showed accelerated aging, with 16 tracts common to both disorders, emphasizing shared and distinct neuroanatomical changes in BD and MDD. These findings suggest that brain aging trajectories could serve as valuable biomarkers for differentiating and understanding the neuropathological underpinnings of BD and MDD as progressive illnesses.

The gray matter models indicated an increased BAG in most brain regions in the BD group, suggesting widespread brain structure degeneration in these individuals. One study found that untreated BD participants had a brain age exceeding their chronological age by 4.28 ± 6.33 years, while those treated with lithium showed an average difference of 0.48 ± 7.60 years (Van Gestel et al., 2019). Another study reported significantly larger BAG in BD participants compared to HCs (van der Markt et al., 2024). Additionally, it was found that the BAG of individuals with BD was approximately 2 years older than that of HCs (Kaufmann et al., 2019). However, other studies found no significant BAG differences in individuals with BD (Hajek et al., 2019; Shahab et al., 2019), and there were observations of significant BAG increases in schizophrenia but not in BD (Nenadić, Dietzek, Langbein, Sauer, & Gaser, 2017).

For MDD, a large-scale Enhancing NeuroImaging Genetics through Meta-Analysis (ENIGMA) study showed that patients with MDD had an increased brain age of +1.08 years compared with controls (Han, Dinga, et al., 2021). These findings were further replicated by the ENIGMA MDD working group, showing a significantly higher estimated brain age than their chronological age, exceeding it by 1 year (Han et al., 2022). These findings were consistent with previous results showing a BAG of +2.78 years (Han, Schnack, et al., 2021) and +2.11 years (Dunlop et al., 2021).

While the findings in the current study are generally consistent with prior studies of BD and MDD, the inconsistent findings in previous brain age research, particularly in BD, may stem from the approach of using entire brain data for modeling, potentially missing region-specific degeneration. The current study’s region-focused brain age prediction approach identified brain regions with accelerated aging in BD and could serve as a biomarker, aiding in the development of future therapeutic strategies.

Our results showed that 13 of the 20 brain regions with the most significant deterioration were common to both the BD and MDD groups, suggesting shared neuropathological mechanisms. Both BD and MDD groups had smaller brain regions in the dorsomedial prefrontal cortex, ventromedial prefrontal cortex, anterior cingulate cortex, and insula (Wise et al., 2017). Compared to HCs, schizophrenia, BD, and MDD groups shared significant gray matter volume reductions in the temporal pole, orbital frontal cortex, insula, parahippocampal gyri, cingulate gyri, dorsolateral prefrontal cortex, angular gyri, and cuneus (Chang et al., 2018). A meta-analysis revealed that BAG varied across psychiatric disorders, with schizophrenia having the largest BAG, followed by BD and then MDD (Ballester et al., 2022). This suggests that different psychiatric disorders are characterized by different degrees of brain degeneration. Our study extended this understanding by quantitatively evaluating the differences in degeneration between different brain regions in affective disorders.

Notably, individuals with BD exhibited significant aging exclusively in several key brain regions, including the right inferior frontal gyrus (operculum), left hippocampus, left amygdala, and right superior parietal gyrus. These regions have consistently shown abnormalities in previous brain imaging studies of individuals with BD (Chen et al., 2022; Hajek, Kopecek, Höschl, & Alda, 2012; Usher, Leucht, Falkai, & Scherk, 2010; Zhang et al., 2021). Subcortical regions, including the striatum, amygdala, and hippocampus, may show differential effects in BD and MDD (Konarski et al., 2008). Moreover, our study identified structural abnormalities in unique brain regions of the MDD group, especially in the left anterior cingulate and paracingulate gyri and bilateral superior frontal gyrus (orbital). These regions have been previously identified as exhibiting significant abnormalities in brain imaging studies of individuals with MDD (Lai, Payne, Byrum, Steffens, & Krishnan, 2000; Liu et al., 2024a,b). Gray matter volume was reduced in the left superior frontal gyrus and left anterior cingulate cortex in patients with MDD compared with those with BD (Chen et al., 2018). These findings suggest that distinct brain regions are affected differently in BD and MDD, highlighting the potential for region-specific biomarkers to identify key brain dysfunction in both disorders.

In the models for the standard deviation map, our findings revealed that the BD group showed accelerated brain aging in 17 brain regions, whereas no comparable results were observed in the MDD group. Our previous study used functional connectivity maps to develop brain age prediction models for individuals with schizophrenia (Zhu, Wu, et al., 2023). However, prior research indicated that blood oxygen level-dependent (BOLD) signal variability had a strong correlation with age and differed significantly from average BOLD signals (Garrett, Kovacevic, McIntosh, & Grady, 2010). Thus, we used standard deviation maps to train brain age prediction models and identified brain regions with accelerated aging in BD. Future studies could integrate diverse BOLD signal characteristics to develop more comprehensive brain age prediction models, enhancing our understanding of how mental illness impacts the degeneration of brain function.

Our findings revealed that both BD and MDD groups exhibited deviations in brain aging trajectories across most white matter tracts in the 48 models for the fractional anisotropy map. Among the top 20 tracts showing the most significant accelerated aging, 16 were shared between BD and MDD. Research has consistently shown white matter abnormalities in both disorders, particularly in the corpus callosum and corona radiata (Cui et al., 2020; Wise et al., 2016). These overlapping tracts suggest common microstructural alterations, indicating similar underlying pathological processes. Further investigation is needed to clarify the specific biological changes driving these alterations and to explore therapeutic potentials. Additionally, our research found disease-specific accelerated aging in the right anterior corona radiata in BD, consistent with previous studies (Karababa et al., 2015; Pavuluri et al., 2009; Sarıçiçek et al., 2016). For MDD, we observed significantly larger BAG in the bilateral retrolenticular part of the internal capsule, unique to MDD and consistent with earlier research (van Velzen et al., 2020; Xiao, He, McWhinnie, & Yao, 2015). These findings suggest distinct pathological processes in BD and MDD, with specific white matter alterations highlighting subtle differences in their neuropathologies.

We found no statistically significant correlations between the BAG and any clinical assessment scores or duration of illness in either group, with one exception: a significant negative correlation was observed between BAG derived from the fractional anisotropy map in the left anterior limb of the internal capsule and MMSE scores in the BD group. The anterior limb of the internal capsule has been widely implicated in BD, and previous studies have demonstrated that alterations in its structural integrity are common among individuals with BD. These changes have been associated with impairments in mood regulation and cognitive functions, including motivation, cognitive processing, and decision-making (Lu et al., 2012; Safadi et al., 2018). Similar to these findings, our finding revealed that greater BAG in the left anterior limb of the internal capsule was associated with lower MMSE scores in the BD group, suggesting that accelerated aging in this region may contribute to reduced cognitive performance. However, no significant correlations were found between MMSE scores and BAG in other brain regions exhibiting accelerated aging. One possible explanation is that the MMSE may lack the sensitivity to detect subtle cognitive impairments, particularly due to ceiling effects (Zadikoff et al., 2008). Future studies employing a broader and more sensitive range of cognitive assessments may provide a more comprehensive understanding of cognitive functioning in individuals with BD and MDD. For severity of symptoms, neither the BD nor the MDD groups reached statistical significance after adjusting for FDR correction. Two possible explanations for these findings were considered. First, the severity of symptoms among the participants with affective disorders included in this study was stable, which may have influenced the observed results. Second, the brain age model constructed from the selected features in this study may not accurately reflect the observed clinical manifestations. Regarding duration of illness, data were available for a subset of participants – specifically, 92 individuals with BD and 38 individuals with MDD. After excluding cases with missing data, partial correlation analyses were performed. However, no significant correlations were observed after applying FDR correction. Several factors may help explain the absence of significant findings. First, the relatively small sample size of the MDD group with available duration data (*n* = 38) likely limited statistical power, reducing the ability to detect meaningful associations. Second, the considerable heterogeneity in illness trajectories may have diluted potential effects. Most individuals with BD were in a chronic stage, with an average illness duration of approximately 20 years. Notably, 80% had experienced illness for more than a decade, and over half (52%) had durations exceeding 20 years. In contrast, 79% of participants with MDD had illness durations under 10 years, reflecting a predominance of earlier-stage cases in this group. Third, differences in treatment histories and the cross-sectional design of the study may have further obscured potential associations between BAG and illness duration.

Some limitations need to be addressed in this study. First, the training dataset included HCs from various age groups, but the cross-sectional design has inherent limitations. Future research should include larger sample sizes and longitudinal data to improve model performance and more accurately reflect the dynamic nature of disease trajectories. This approach may help to further clarify the relationship between the BAG and disease progression. Additionally, studies with larger and more balanced sex-specific samples are warranted to support the development of sex-stratified brain age prediction models and to explore potential sex-related differences in brain aging trajectories. Second, the sample sizes for the MDD and BD groups were limited, indicating the need for larger samples or multiple cohorts in future research to validate the findings. Third, the data used in this study were derived from the TAMI cohort, which did not include documentation of participants’ mood states at the time of MRI acquisition. As a result, we could not determine their precise clinical status during scanning. Given that the current mood state may influence both resting-state functional activity and brain age predictions, this represents a potential source of unaccounted variability. Future prospective studies should include current mood assessments during MRI scanning – either as variables of interest or as covariates – to more accurately evaluate their impact on BAG estimates. Finally, the absence of detailed medication data for BD and MDD participants precluded an in-depth analysis of medication effects. Future research should include medication data or recruit drug-naive participants to better examine the impact of affective disorders on the brain.

This study demonstrated that region-specific brain aging trajectory models performed robustly, identifying accelerated aging in various gray matter regions in both BD and MDD. Notably, several highly deteriorated regions were common to both disorders, indicating shared neuropathological mechanisms. The BD group showed accelerated aging in multiple brain regions on standard deviation maps, while no such regions were found in MDD. Additionally, fractional anisotropy analysis revealed aging white matter tracts in both BD and MDD, with several tracts common to both disorders. Importantly, there were also unique brain regions with accelerated aging specific to each disorder, highlighting distinct neuropathological processes in BD and MDD. These findings highlight the potential of brain aging trajectories as biomarkers, offering insights into distinct and overlapping neuroanatomical changes in BD and MDD. Identifying key areas of brain dysfunction is critical for developing precision diagnosis and noninvasive treatments, such as personalized transcranial magnetic stimulation or deep brain stimulation. Our approach of incorporating region-specific changes in brain structure and function over time could enhance understanding and treatment of mental illness. Future research should include longitudinal data, larger sample sizes, and detailed medication information to improve model performance and clinical correlates of the region-specific brain aging trajectory approach.

## Supporting information

Zhu et al. supplementary material Zhu et al. supplementary material

## Supplementary material

The supplementary material for this article can be found at http://doi.org/10.1017/S0033291725101517.
