# Supplementary material for: Identifying key brain pathology in bipolar and unipolar depression using a region-specific brain aging trajectories approach: Insights from the Taiwan Aging and Mental Illness Cohort
Source: Psychol Med. 2025 Aug 29;55:e253. doi: 10.1017/S0033291725101517 (PMC13040587; doi:10.1017/S0033291725101517)
Supplement: Zhu et al. supplementary material [file S0033291725101517sup001.docx]

**Identifying Key Brain Pathology in Bipolar and Unipolar Depression Using a Region-Specific Brain Aging Trajectories Approach: Insights from the Taiwan Aging and Mental Illness Cohort**

– SUPPLEMENTARY MATERIAL –

Jun-Ding Zhu, I-Jou Chi, Hui-Yun Hsu, Shih-Jen Tsai, Albert C. Yang*

**Supplementary methods**

**Participants**

Participants in this study were sourced from the Taiwan Aging and Mental Illness (TAMI) cohort (Shen, Tsai, Lin, & Yang, 2023; Yang et al., 2015; Yang, Tsai, Lin, Peng, & Huang, 2018; Zhu, Tsai, Lin, Lee, & Yang, 2023; Zhu, Wu, Tsai, Lin, & Yang, 2023). We included 230 healthy controls (HC) aged 20 to 84 for the training dataset and validated the results with an independent test dataset of 110 HCs. The participants included in the training dataset had a mean age of 42.14 ± 16.76 years and a mean education of 15.81 ± 3.67 years. The mean score on the Mini-Mental State Examination (MMSE) was 28.87 ± 1.09. Of the 230 participants, 96 were male and 134 were female. Male participants had a mean age of 39.39 years and a mean of 16.93 years of education, with a mean MMSE score of 28.88. Female participants had a mean age of 44.11 years and a mean of 15.01 years of education, with a comparable mean MMSE score of 28.87 (Supplementary Figure 1). This study also included 110 individuals with bipolar I disorder (BD) as well as 68 individuals with unipolar depression (MDD) from the TAMI cohort. The diagnosis of mental illness was based on the Diagnostic and Statistical Manual of Mental Disorders (DSM-IV-TR). Exclusion criteria of this study included any DSM-IV-TR diagnosis of schizophrenia or other psychoses, intellectual disability, organic mental disorders, autoimmune or immunological diseases, recent substance abuse, current pregnancy or breastfeeding, and unstable physical illness. In addition, the MDD group excluded participants over the age of 65. The test dataset was selected based on the absence of any history of neurological or psychiatric disorders. Two control groups of equal size were randomly selected from the independent test dataset for subsequent comparison based on the sample size and sex ratio of the BD and MDD groups. Demographic and clinical characteristics of participants are shown in Table 1. The MMSE scores were obtained for all participants in the TAMI cohort. Data from clinical assessments, including the Hamilton Depression Rating Scale (HAM-D-21) and the Hamilton Anxiety Rating Scale (HAM-A), were obtained for the MDD group. Scores from the Young Mania Rating Scale (YMRS) and the previously mentioned clinical scales were gathered for the BD group. To determine the correlation between brain age gap and clinical assessment scores, Partial correlation analysis was conducted after excluding individuals with missing data (refer to Table 1 for details). The study was conducted in accordance with the Declaration of Helsinki, and the protocol was approved by the Institutional Review Board of Taipei Veterans General Hospital, Taiwan (2023-12-003A). Obtaining informed consent was exempted by the Institutional Review Board because the data was deidentified in the TAMI cohort.

**Image acquisition**

The magnetic resonance imaging (MRI) data acquisition for all participants in this study was conducted using a 3T MRI scanner (Siemens Magnetom Tim Trio, Erlangen, Germany) equipped with a 12-channel head coil at National Yang Ming Chiao Tung University. The scanning protocols were consistent with those established in previous studies (Shen et al., 2023; Zhu, Tsai, et al., 2023; Zhu, Wu, et al., 2023). For T1-weighted MRI, data acquisition was performed using a sagittal 3D magnetization-prepared rapid gradient echo (MPRAGE) sequence. The parameters used for this sequence were: repetition time (TR) = 2530 ms, echo time (TE) = 3.5 ms, inversion time = 1100 ms, matrix size = 256 × 256, the number of slices = 192, slice thickness = 1 mm, voxel size = 1.0 × 1.0 × 1.0 mm^3^, and a flip angle of 7°. The resting-state functional MRI (fMRI) images were acquired using a T2*-weighted gradient-echo-planar imaging (EPI) sequence with the following settings: The imaging parameters were: TR = 2500 ms, TE = 27 ms, matrix size = 64 × 64, voxel size = 3.4 × 3.4 × 3.4 mm^3,^ total time points = 200, field of view (FOV) = 200 mm, and a flip angle of 77°. DTI images were obtained using a single-shot spin-echo EPI sequence in the axial plane. The imaging parameters were: TR = 11,000 ms, TE = 104 ms, number of excitations = 3, matrix size = 128 × 128, FOV = 26 cm, the number of slices = 70, slice thickness = 2.0 mm, The b-value is 1000 s/mm^2^ with thirty isotropic diffusion directions and three non-diffusion weighted T2 images.

**Image preprocessing**

*Gray matter map construction*

The preprocessing of raw T1-weighted MRI and raw resting-state fMRI data for each participant was performed using Statistical Parametric Mapping (SPM) 12 and the Data Processing & Analysis for Brain Imaging (DPABI) toolbox (Yan, Wang, Zuo, & Zang, 2016), operating within MATLAB R2022a (MathWorks, Natick, MA, USA). The process of raw T1-weighted MRI involved the following steps: (1) the T1-weighted images were manually reoriented based on the anterior commissure-posterior commissure line; (2) all images were normalized to the MNI152 standard space and segmented into gray matter, white matter, and cerebrospinal fluid regions; and (3) the automated anatomical labeling (AAL) atlas was used for further segmentation of the gray matter images, resulting in 90 gray matter maps for each participant (Tzourio-Mazoyer et al., 2002).

*Standard deviation map construction*

The process of raw resting-state fMRI data involved several key steps: (1) the first five data points were removed; (2) slice-timing correction, realignment, and manual reorientation of all images were applied; (3) the reoriented images were coregistered with T1-weighted images; (4) the data was normalized to the MNI152 standard space and resampled to a voxel size of 3 × 3 × 3 mm³; (5) covariates, including those related to head motions, white matter, and cerebrospinal fluid, were regressed; (6) temporal lowpass filtering was executed within the range of 0.01-0.1 Hz; (7) the voxel-wise standard deviation of the blood-oxygen-level-dependent signal of each voxel was calculated; and (8) ninety standard deviation maps for each participant were obtained using the AAL atlas (Tzourio-Mazoyer et al., 2002).

*Fractional anisotropy map construction*

The fractional anisotropy map was constructed using FMRIB Software Library v6.0 (FSL) (Jenkinson, Beckmann, Behrens, Woolrich, & Smith, 2012). The process involved the following steps: (1) corrected eddy currents and movements in the raw DTI data using the eddy tool; (2) extracted brain tissue and removed non-brain tissue using the brain extraction tool; (3) created fractional anisotropy images by fitting the eddy-corrected data into a tensor model at each voxel; (4) registered and aligned all fractional anisotropy images using the FMRIB58_FA standard-space image as the target; (5) normalized all fractional anisotropy images to the MNI152 standard space; and (6) the JHU-ICBM-Labels-1mm atlas was used to segment into 48 fractional anisotropy maps for each participant (Hua et al., 2008; Wakana et al., 2007).

**Feature selection**

This study used a systematic approach to identifying key features (i.e., voxels) that exhibit the strongest correlations with chronological age across different brain regions (Figure 1B). The process was applied to all 228 maps, including 90 gray matter, 90 standard deviation, and 48 fractional anisotropy maps. Initially, 70% of the participants from the training dataset were randomly selected to calculate Pearson's correlation coefficient. This was done to evaluate the relationship between each voxel in different brain regions and chronological age. To ensure robustness, this step was iteratively conducted 1000 times. Subsequently, the top 50% of voxels exhibiting the highest correlation coefficient values with chronological age were identified in these 1000 trials. The objective of this process was to refine and pinpoint the most relevant key voxels within each brain region. To reinforce the selection process, the initial two steps were replicated 100 times, generating 100 different sets of key voxels. Each voxel selected from these sets was then used as a key feature for model training. The repeated iteration was necessary to ensure the consistency and reliability of the selected voxels. This rigorous and comprehensive feature selection process established a set of key features for each of the 90 gray matter, 90 standard deviation, and 48 fractional anisotropy maps. These features were then used to construct a brain-age prediction model for different brain regions (Zhu et al., 2022; Zhu, Wu, et al., 2023) .

**Supplementary results**

*Brain-age prediction model performance*

Ninety models for gray matter, 90 for standard deviation, and 48 for fractional anisotropy maps were developed using Gaussian process regression with five-fold cross-validation. The models for gray matter map demonstrated consistent mean absolute errors (MAEs) and robust correlations before and after bias correction across 90 brain regions. Before bias correction, the average MAE and average correlation coefficient for the training dataset were 9.38 ± 1.06 years and 0.71 ± 0.07, respectively, while for the test dataset, they were 9.21 ± 1.03 and 0.60 ± 0.09, respectively. After bias correction, the training dataset had the average MAE of 6.32 ± 0.30 and the average correlation coefficient of 0.90 ± 0.01. The test dataset had the average MAE of 6.71 ± 0.66 and the average correlation coefficient of 0.84 ± 0.03, as detailed in Supplementary Table 1.

Similarly, the 90 models for standard deviation map displayed consistent MAEs and correlations between the training and test datasets across various brain regions. Before bias correction, the average MAE and the average correlation coefficient for the training dataset were 13.31 ± 0.79 years and 0.39 ± 0.09, respectively. For the test dataset, the corresponding values were 11.36 ± 0.55 and 0.23 ± 0.12. After correcting for bias, the training dataset showed the average MAE of 4.60 ± 0.74 and the average correlation coefficient of 0.95 ± 0.02. The test dataset had the average MAE of 4.91 ± 1.01 and the average correlation coefficient of 0.90 ± 0.04, as detailed in Supplementary Table 2.

The results of the 48 models for fractional anisotropy map revealed stable MAEs and significant linear relationships between brain ages and chronological ages. Before bias correction, the average MAE and the average correlation coefficient for the training dataset were 10.66 ± 1.28 years and 0.64 ± 0.09, respectively. For the test dataset, these values were 9.98 ± 1.08 and 0.58 ± 0.11. After correcting for bias, the training dataset had the average MAE of 6.20 ± 0.34 and the average correlation coefficient of 0.91 ± 0.01. The test dataset had the average MAE of 6.64 ± 0.73 and the average correlation coefficient of 0.87 ± 0.03. Supplementary Table 3 provides additional details. These findings demonstrated that the brain-age prediction models developed in this study consistently performed well and were stable on both the training and test datasets.


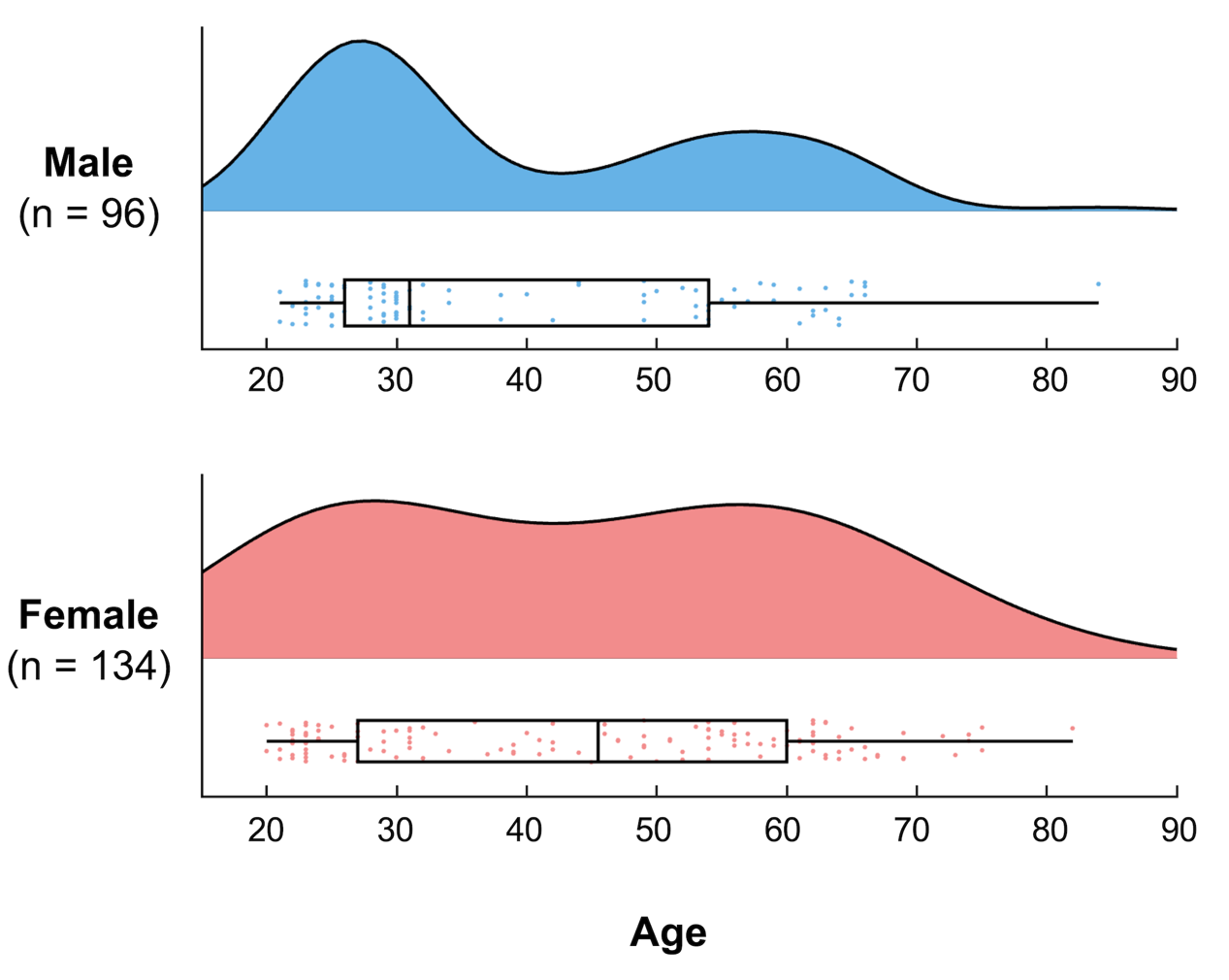


**Supplementary Figure 1. Age distribution by sex in the training dataset.** This figure shows the age distribution of the training dataset, stratified by sex. The top panel represents male participants (n = 96), while the bottom panel represents female participants (n = 134).

**Supplementary Table 1. Model performances of 90 models for gray matter map in training and test datasets.**

| **AAL label number** | **Regions** | **Left/Right** | **Before bias correction** | | | |  | **After bias correction** | | | |
| --- | --- | --- | --- | --- | --- | --- | --- | --- | --- | --- | --- |
|  |  |  | **Training dataset** | | **Test dataset** | |  | **Training dataset** | | **Test dataset** | |
|  |  |  | **MAE (years)** | **correlation coefficient** | **MAE (years)** | **correlation coefficient** |  | **MAE (years)** | **correlation coefficient** | **MAE (years)** | **correlation coefficient** |
| 1 | Precental gyrus | Left | 9.17 | 0.74 | 8.18 | 0.69 |  | 6.45 | 0.90 | 5.35 | 0.88 |
| 2 | Precental gyrus | Right | 8.23 | 0.79 | 8.12 | 0.71 |  | 6.04 | 0.90 | 6.12 | 0.86 |
| 3 | Superior frontal gyrus, dorsolateral | Left | 8.86 | 0.75 | 9.11 | 0.66 |  | 6.31 | 0.90 | 6.44 | 0.85 |
| 4 | Superior frontal gyrus, dorsolateral | Right | 8.39 | 0.78 | 9.41 | 0.67 |  | 6.14 | 0.90 | 7.26 | 0.83 |
| 5 | Superior frontal gyrus, orbital part | Left | 10.14 | 0.67 | 9.63 | 0.61 |  | 6.55 | 0.90 | 6.48 | 0.86 |
| 6 | Superior frontal gyrus, orbital part | Right | 10.13 | 0.68 | 10.94 | 0.48 |  | 6.46 | 0.90 | 7.59 | 0.80 |
| 7 | Middle frontal gyrus | Left | 9.07 | 0.73 | 9.89 | 0.56 |  | 6.32 | 0.90 | 7.24 | 0.81 |
| 8 | Middle frontal gyrus | Right | 8.95 | 0.74 | 9.22 | 0.62 |  | 6.27 | 0.90 | 6.93 | 0.83 |
| 9 | Middle frontal gyrus, orbital part | Left | 11.18 | 0.58 | 9.80 | 0.54 |  | 6.17 | 0.91 | 5.85 | 0.87 |
| 10 | Middle frontal gyrus, orbital part | Right | 11.02 | 0.62 | 10.34 | 0.54 |  | 6.36 | 0.90 | 7.41 | 0.84 |
| 11 | Inferior frontal gyrus, opercular part | Left | 9.18 | 0.72 | 9.50 | 0.57 |  | 6.36 | 0.90 | 6.61 | 0.83 |
| 12 | Inferior frontal gyrus, opercular part | Right | 8.63 | 0.76 | 9.36 | 0.62 |  | 6.23 | 0.90 | 6.85 | 0.82 |
| 13 | Inferior frontal gyrus, triangular part | Left | 9.17 | 0.72 | 9.88 | 0.51 |  | 6.46 | 0.90 | 7.40 | 0.79 |
| 14 | Inferior frontal gyrus, triangular part | Right | 9.58 | 0.69 | 10.83 | 0.46 |  | 6.46 | 0.90 | 8.03 | 0.77 |
| 15 | Inferior frontal gyrus, orbital part | Left | 9.43 | 0.71 | 8.90 | 0.64 |  | 6.62 | 0.90 | 7.03 | 0.84 |
| 16 | Inferior frontal gyrus, orbital part | Right | 9.42 | 0.71 | 8.69 | 0.64 |  | 6.66 | 0.89 | 6.66 | 0.84 |
| 17 | Rolandic operculum | Left | 9.62 | 0.69 | 7.83 | 0.72 |  | 6.33 | 0.90 | 5.94 | 0.89 |
| 18 | Rolandic operculum | Right | 9.32 | 0.72 | 8.97 | 0.67 |  | 6.50 | 0.90 | 6.64 | 0.86 |
| 19 | Supplementary motor area | Left | 9.74 | 0.70 | 9.27 | 0.62 |  | 6.74 | 0.90 | 6.69 | 0.85 |
| 20 | Supplementary motor area | Right | 9.59 | 0.71 | 8.59 | 0.63 |  | 6.36 | 0.90 | 6.77 | 0.84 |
| 21 | Olfactory cortex | Left | 9.08 | 0.74 | 8.74 | 0.68 |  | 6.45 | 0.90 | 6.37 | 0.86 |
| 22 | Olfactory cortex | Right | 9.18 | 0.73 | 8.97 | 0.57 |  | 6.34 | 0.90 | 6.22 | 0.83 |
| 23 | Superior frontal gyrus, medial | Left | 8.19 | 0.78 | 8.76 | 0.65 |  | 6.17 | 0.90 | 7.01 | 0.82 |
| 24 | Superior frontal gyrus, medial | Right | 8.91 | 0.74 | 9.22 | 0.64 |  | 6.63 | 0.90 | 6.83 | 0.84 |
| 25 | Superior frontal gyrus, medial orbital | Left | 9.55 | 0.72 | 9.62 | 0.57 |  | 6.49 | 0.90 | 7.61 | 0.80 |
| 26 | Superior frontal gyrus, medial orbital | Right | 9.22 | 0.71 | 9.40 | 0.60 |  | 6.58 | 0.90 | 6.86 | 0.83 |
| 27 | Gyrus rectus | Left | 10.29 | 0.66 | 9.23 | 0.60 |  | 6.32 | 0.90 | 6.57 | 0.86 |
| 28 | Gyrus rectus | Right | 8.97 | 0.75 | 10.25 | 0.57 |  | 6.27 | 0.90 | 7.79 | 0.80 |
| 29 | Insula | Left | 8.33 | 0.76 | 7.95 | 0.72 |  | 6.47 | 0.90 | 6.30 | 0.87 |
| 30 | Insula | Right | 7.93 | 0.78 | 7.89 | 0.71 |  | 6.01 | 0.90 | 6.21 | 0.85 |
| 31 | Anterior cingulate and paracingulate gyri | Left | 9.01 | 0.74 | 9.69 | 0.55 |  | 6.48 | 0.90 | 7.12 | 0.80 |
| 32 | Anterior cingulate and paracingulate gyri | Right | 9.72 | 0.69 | 8.73 | 0.65 |  | 6.03 | 0.90 | 6.13 | 0.87 |
| 33 | Median cingulate and paracingulate gyri | Left | 9.76 | 0.69 | 9.25 | 0.59 |  | 6.36 | 0.90 | 6.12 | 0.85 |
| 34 | Median cingulate and paracingulate gyri | Right | 9.89 | 0.70 | 8.47 | 0.65 |  | 6.15 | 0.90 | 5.23 | 0.89 |
| 35 | Posterior cingulate gyrus | Left | 12.21 | 0.50 | 11.30 | 0.48 |  | 5.63 | 0.92 | 5.93 | 0.88 |
| 36 | Posterior cingulate gyrus | Right | 13.35 | 0.38 | 11.53 | 0.28 |  | 4.57 | 0.95 | 4.41 | 0.92 |
| 37 | Hippocampus | Left | 7.48 | 0.82 | 7.25 | 0.75 |  | 6.07 | 0.91 | 6.14 | 0.86 |
| 38 | Hippocampus | Right | 8.21 | 0.80 | 8.49 | 0.71 |  | 6.27 | 0.90 | 7.38 | 0.84 |
| 39 | Parahippocampal gyrus | Left | 8.36 | 0.74 | 8.22 | 0.72 |  | 6.58 | 0.89 | 6.58 | 0.87 |
| 40 | Parahippocampal gyrus | Right | 8.74 | 0.75 | 8.74 | 0.63 |  | 6.35 | 0.90 | 6.62 | 0.83 |
| 41 | Amygdala | Left | 8.11 | 0.79 | 8.61 | 0.68 |  | 6.36 | 0.90 | 7.20 | 0.83 |
| 42 | Amygdala | Right | 8.83 | 0.73 | 8.73 | 0.61 |  | 6.51 | 0.89 | 7.16 | 0.82 |
| 43 | Calcarine fissure and surrounding cortex | Left | 10.83 | 0.65 | 9.67 | 0.58 |  | 6.06 | 0.91 | 6.76 | 0.85 |
| 44 | Calcarine fissure and surrounding cortex | Right | 10.77 | 0.64 | 9.91 | 0.57 |  | 6.17 | 0.91 | 5.96 | 0.86 |
| 45 | Cuneus | Left | 10.88 | 0.63 | 10.12 | 0.50 |  | 6.36 | 0.90 | 6.70 | 0.83 |
| 46 | Cuneus | Right | 10.50 | 0.64 | 9.57 | 0.57 |  | 6.64 | 0.90 | 6.54 | 0.85 |
| 47 | Lingual gyrus | Left | 8.11 | 0.77 | 8.61 | 0.64 |  | 6.03 | 0.90 | 6.32 | 0.83 |
| 48 | Lingual gyrus | Right | 8.72 | 0.76 | 8.72 | 0.67 |  | 6.09 | 0.90 | 7.04 | 0.84 |
| 49 | Superior occipital gyrus | Left | 10.53 | 0.64 | 11.11 | 0.49 |  | 6.29 | 0.90 | 6.77 | 0.83 |
| 50 | Superior occipital gyrus | Right | 10.18 | 0.66 | 9.54 | 0.55 |  | 6.23 | 0.90 | 6.26 | 0.85 |
| 51 | Middle occipital gyrus | Left | 9.43 | 0.72 | 8.99 | 0.61 |  | 6.33 | 0.90 | 6.69 | 0.84 |
| 52 | Middle occipital gyrus | Right | 9.90 | 0.69 | 9.05 | 0.61 |  | 6.59 | 0.90 | 6.38 | 0.85 |
| 53 | Inferior occipital gyrus | Left | 10.45 | 0.66 | 9.46 | 0.59 |  | 6.54 | 0.90 | 7.53 | 0.84 |
| 54 | Inferior occipital gyrus | Right | 10.42 | 0.65 | 10.17 | 0.50 |  | 6.44 | 0.90 | 7.29 | 0.82 |
| 55 | Fusiform gyrus | Left | 9.70 | 0.69 | 9.81 | 0.59 |  | 6.36 | 0.90 | 7.40 | 0.83 |
| 56 | Fusiform gyrus | Right | 9.89 | 0.68 | 9.80 | 0.50 |  | 6.37 | 0.90 | 7.24 | 0.81 |
| 57 | Postcentral gyrus | Left | 9.00 | 0.75 | 8.08 | 0.70 |  | 6.20 | 0.91 | 6.24 | 0.87 |
| 58 | Postcentral gyrus | Right | 8.57 | 0.76 | 8.24 | 0.69 |  | 6.27 | 0.90 | 6.32 | 0.86 |
| 59 | Superior parietal gyrus | Left | 9.79 | 0.69 | 10.69 | 0.52 |  | 6.35 | 0.90 | 7.59 | 0.80 |
| 60 | Superior parietal gyrus | Right | 9.71 | 0.71 | 9.91 | 0.57 |  | 6.56 | 0.90 | 6.71 | 0.83 |
| 61 | Inferior parietal, but supramarginal and angular gyri | Left | 9.27 | 0.73 | 9.98 | 0.57 |  | 6.24 | 0.90 | 7.20 | 0.81 |
| 62 | Inferior parietal, but supramarginal and angular gyri | Right | 8.80 | 0.77 | 10.22 | 0.54 |  | 6.33 | 0.91 | 8.61 | 0.76 |
| 63 | Supramarginal gyrus | Left | 9.89 | 0.69 | 10.49 | 0.50 |  | 6.28 | 0.91 | 7.22 | 0.80 |
| 64 | Supramarginal gyrus | Right | 9.94 | 0.70 | 9.58 | 0.58 |  | 6.01 | 0.91 | 6.87 | 0.84 |
| 65 | Angular gyrus | Left | 10.90 | 0.64 | 10.12 | 0.54 |  | 6.13 | 0.91 | 6.92 | 0.84 |
| 66 | Angular gyrus | Right | 8.91 | 0.75 | 9.25 | 0.56 |  | 6.36 | 0.90 | 6.97 | 0.80 |
| 67 | Precuneus | Left | 9.52 | 0.73 | 9.56 | 0.57 |  | 6.62 | 0.90 | 6.97 | 0.81 |
| 68 | Precuneus | Right | 9.06 | 0.74 | 9.20 | 0.58 |  | 6.21 | 0.90 | 6.75 | 0.81 |
| 69 | Paracentral lobule | Left | 9.67 | 0.70 | 9.34 | 0.64 |  | 6.61 | 0.90 | 7.12 | 0.85 |
| 70 | Paracentral lobule | Right | 9.98 | 0.68 | 9.95 | 0.49 |  | 6.68 | 0.90 | 6.58 | 0.82 |
| 71 | Caudate nucleus | Left | 8.52 | 0.75 | 8.05 | 0.71 |  | 6.62 | 0.89 | 6.09 | 0.87 |
| 72 | Caudate nucleus | Right | 7.83 | 0.79 | 7.77 | 0.72 |  | 6.20 | 0.90 | 6.30 | 0.85 |
| 73 | Lenticular nucleus putamen | Left | 8.99 | 0.73 | 7.20 | 0.71 |  | 6.78 | 0.89 | 5.70 | 0.88 |
| 74 | Lenticular nucleus putamen | Right | 8.15 | 0.77 | 8.26 | 0.61 |  | 6.40 | 0.90 | 6.02 | 0.83 |
| 75 | Lenticular nucleus, pallidum | Left | 9.59 | 0.69 | 9.80 | 0.48 |  | 6.09 | 0.91 | 6.47 | 0.83 |
| 76 | Lenticular nucleus, pallidum | Right | 9.98 | 0.66 | 9.35 | 0.48 |  | 6.54 | 0.90 | 7.04 | 0.82 |
| 77 | Thalamus | Left | 6.34 | 0.88 | 5.70 | 0.86 |  | 5.53 | 0.92 | 5.33 | 0.90 |
| 78 | Thalamus | Right | 6.63 | 0.87 | 6.37 | 0.84 |  | 5.74 | 0.92 | 5.26 | 0.90 |
| 79 | Heschl gyrus | Left | 9.99 | 0.67 | 8.33 | 0.67 |  | 6.30 | 0.90 | 6.35 | 0.88 |
| 80 | Heschl gyrus | Right | 9.87 | 0.69 | 8.85 | 0.67 |  | 6.57 | 0.90 | 6.81 | 0.87 |
| 81 | Superior temporal gyrus | Left | 9.06 | 0.73 | 7.92 | 0.72 |  | 6.48 | 0.90 | 6.46 | 0.88 |
| 82 | Superior temporal gyrus | Right | 9.13 | 0.74 | 8.82 | 0.66 |  | 6.14 | 0.90 | 6.76 | 0.85 |
| 83 | Temporal pole: superior temporal gyrus | Left | 8.28 | 0.78 | 8.59 | 0.70 |  | 6.43 | 0.90 | 7.01 | 0.84 |
| 84 | Temporal pole: superior temporal gyrus | Right | 8.30 | 0.77 | 9.15 | 0.66 |  | 6.50 | 0.90 | 7.54 | 0.83 |
| 85 | Middle temporal gyrus | Left | 8.93 | 0.75 | 9.39 | 0.61 |  | 6.33 | 0.90 | 6.69 | 0.83 |
| 86 | Middle temporal gyrus | Right | 9.15 | 0.73 | 9.75 | 0.59 |  | 6.49 | 0.90 | 7.41 | 0.81 |
| 87 | Temporal pole: middle temporal gyrus | Left | 11.07 | 0.60 | 11.48 | 0.34 |  | 6.16 | 0.91 | 7.41 | 0.79 |
| 88 | Temporal pole: middle temporal gyrus | Right | 10.53 | 0.65 | 11.21 | 0.41 |  | 6.36 | 0.90 | 7.28 | 0.78 |
| 89 | Inferior temporal gyrus | Left | 9.75 | 0.70 | 9.33 | 0.62 |  | 6.72 | 0.90 | 6.79 | 0.84 |
| 90 | Inferior temporal gyrus | Right | 8.92 | 0.75 | 8.97 | 0.62 |  | 6.61 | 0.90 | 7.44 | 0.82 |

Abbreviations: AAL, automated anatomical labeling; MAE, mean absolute error.

**Supplementary Table 2. Model performances of 90 models for standard deviation map in training and test datasets.**

| **AAL label number** | **Regions** | **Left/Right** | **Before bias correction** | | | |  | **After bias correction** | | | |
| --- | --- | --- | --- | --- | --- | --- | --- | --- | --- | --- | --- |
|  |  |  | **Training dataset** | | **Test dataset** | |  | **Training dataset** | | **Test dataset** | |
|  |  |  | **MAE (years)** | **correlation coefficient** | **MAE (years)** | **correlation coefficient** |  | **MAE (years)** | **correlation coefficient** | **MAE (years)** | **correlation coefficient** |
| 1 | Precental gyrus | Left | 13.45 | 0.38 | 11.50 | 0.25 |  | 4.82 | 0.94 | 5.23 | 0.90 |
| 2 | Precental gyrus | Right | 13.63 | 0.37 | 11.49 | 0.18 |  | 4.43 | 0.95 | 4.94 | 0.90 |
| 3 | Superior frontal gyrus, dorsolateral | Left | 14.00 | 0.30 | 11.80 | 0.11 |  | 3.78 | 0.96 | 4.31 | 0.92 |
| 4 | Superior frontal gyrus, dorsolateral | Right | 13.64 | 0.37 | 11.86 | 0.06 |  | 4.31 | 0.95 | 4.80 | 0.90 |
| 5 | Superior frontal gyrus, orbital part | Left | 13.70 | 0.35 | 12.66 | 0.04 |  | 4.26 | 0.95 | 5.20 | 0.88 |
| 6 | Superior frontal gyrus, orbital part | Right | 13.47 | 0.38 | 11.78 | 0.04 |  | 4.65 | 0.95 | 5.11 | 0.88 |
| 7 | Middle frontal gyrus | Left | 13.54 | 0.35 | 11.71 | 0.16 |  | 4.23 | 0.95 | 4.60 | 0.90 |
| 8 | Middle frontal gyrus | Right | 13.13 | 0.42 | 11.97 | 0.18 |  | 4.81 | 0.94 | 5.81 | 0.86 |
| 9 | Middle frontal gyrus, orbital part | Left | 14.44 | 0.23 | 12.16 | 0.05 |  | 3.22 | 0.97 | 3.14 | 0.96 |
| 10 | Middle frontal gyrus, orbital part | Right | 14.40 | 0.25 | 11.47 | 0.09 |  | 3.59 | 0.97 | 3.65 | 0.94 |
| 11 | Inferior frontal gyrus, opercular part | Left | 12.50 | 0.47 | 11.72 | 0.17 |  | 5.46 | 0.93 | 6.44 | 0.82 |
| 12 | Inferior frontal gyrus, opercular part | Right | 13.23 | 0.42 | 11.81 | 0.17 |  | 5.02 | 0.94 | 5.25 | 0.88 |
| 13 | Inferior frontal gyrus, triangular part | Left | 12.34 | 0.48 | 11.40 | 0.24 |  | 5.24 | 0.93 | 5.91 | 0.87 |
| 14 | Inferior frontal gyrus, triangular part | Right | 13.31 | 0.41 | 10.86 | 0.24 |  | 5.03 | 0.94 | 5.51 | 0.88 |
| 15 | Inferior frontal gyrus, orbital part | Left | 12.83 | 0.47 | 11.58 | 0.27 |  | 4.67 | 0.94 | 4.87 | 0.90 |
| 16 | Inferior frontal gyrus, orbital part | Right | 12.98 | 0.45 | 10.73 | 0.29 |  | 5.04 | 0.94 | 5.80 | 0.87 |
| 17 | Rolandic operculum | Left | 13.68 | 0.36 | 12.43 | 0.04 |  | 4.63 | 0.95 | 5.16 | 0.88 |
| 18 | Rolandic operculum | Right | 13.88 | 0.33 | 11.56 | 0.10 |  | 4.32 | 0.96 | 4.50 | 0.92 |
| 19 | Supplementary motor area | Left | 13.89 | 0.33 | 11.27 | 0.20 |  | 4.33 | 0.95 | 4.25 | 0.93 |
| 20 | Supplementary motor area | Right | 13.17 | 0.40 | 11.64 | 0.21 |  | 4.74 | 0.94 | 5.53 | 0.87 |
| 21 | Olfactory cortex | Left | 13.10 | 0.43 | 11.40 | 0.39 |  | 4.88 | 0.94 | 5.04 | 0.91 |
| 22 | Olfactory cortex | Right | 12.97 | 0.43 | 11.31 | 0.24 |  | 5.03 | 0.94 | 4.86 | 0.89 |
| 23 | Superior frontal gyrus, medial | Left | 13.82 | 0.35 | 11.00 | 0.32 |  | 4.17 | 0.96 | 4.36 | 0.93 |
| 24 | Superior frontal gyrus, medial | Right | 14.10 | 0.31 | 11.26 | 0.20 |  | 3.55 | 0.96 | 4.27 | 0.93 |
| 25 | Superior frontal gyrus, medial orbital | Left | 13.76 | 0.35 | 11.06 | 0.22 |  | 4.03 | 0.96 | 4.65 | 0.92 |
| 26 | Superior frontal gyrus, medial orbital | Right | 14.23 | 0.28 | 10.92 | 0.25 |  | 3.63 | 0.97 | 4.23 | 0.94 |
| 27 | Gyrus rectus | Left | 14.40 | 0.23 | 12.10 | 0.07 |  | 3.31 | 0.97 | 3.41 | 0.95 |
| 28 | Gyrus rectus | Right | 14.28 | 0.24 | 11.75 | 0.20 |  | 3.64 | 0.97 | 3.66 | 0.95 |
| 29 | Insula | Left | 12.26 | 0.52 | 10.59 | 0.39 |  | 5.30 | 0.93 | 4.94 | 0.89 |
| 30 | Insula | Right | 11.53 | 0.57 | 9.95 | 0.47 |  | 5.69 | 0.92 | 6.32 | 0.86 |
| 31 | Anterior cingulate and paracingulate gyri | Left | 12.94 | 0.45 | 10.52 | 0.37 |  | 5.27 | 0.93 | 6.01 | 0.87 |
| 32 | Anterior cingulate and paracingulate gyri | Right | 12.35 | 0.49 | 10.56 | 0.31 |  | 5.28 | 0.93 | 5.74 | 0.86 |
| 33 | Median cingulate and paracingulate gyri | Left | 13.57 | 0.39 | 11.14 | 0.23 |  | 4.85 | 0.95 | 5.32 | 0.89 |
| 34 | Median cingulate and paracingulate gyri | Right | 13.14 | 0.43 | 11.12 | 0.22 |  | 5.07 | 0.94 | 5.90 | 0.87 |
| 35 | Posterior cingulate gyrus | Left | 13.50 | 0.37 | 10.94 | 0.30 |  | 4.71 | 0.95 | 4.79 | 0.91 |
| 36 | Posterior cingulate gyrus | Right | 13.89 | 0.36 | 11.16 | 0.15 |  | 4.36 | 0.95 | 5.01 | 0.90 |
| 37 | Hippocampus | Left | 14.42 | 0.27 | 11.31 | 0.17 |  | 3.39 | 0.97 | 3.24 | 0.95 |
| 38 | Hippocampus | Right | 13.01 | 0.43 | 11.78 | 0.19 |  | 4.86 | 0.94 | 4.87 | 0.88 |
| 39 | Parahippocampal gyrus | Left | 14.10 | 0.29 | 11.09 | 0.38 |  | 4.04 | 0.96 | 3.81 | 0.95 |
| 40 | Parahippocampal gyrus | Right | 13.43 | 0.37 | 10.74 | 0.32 |  | 4.82 | 0.94 | 4.28 | 0.92 |
| 41 | Amygdala | Left | 13.14 | 0.41 | 12.59 | 0.03 |  | 4.42 | 0.95 | 5.21 | 0.87 |
| 42 | Amygdala | Right | 14.00 | 0.29 | 11.91 | 0.11 |  | 4.00 | 0.96 | 3.75 | 0.93 |
| 43 | Calcarine fissure and surrounding cortex | Left | 13.18 | 0.41 | 11.39 | 0.21 |  | 4.56 | 0.95 | 4.52 | 0.91 |
| 44 | Calcarine fissure and surrounding cortex | Right | 13.45 | 0.38 | 11.35 | 0.18 |  | 4.34 | 0.96 | 4.46 | 0.91 |
| 45 | Cuneus | Left | 13.24 | 0.41 | 11.99 | 0.17 |  | 5.21 | 0.94 | 5.52 | 0.87 |
| 46 | Cuneus | Right | 12.92 | 0.45 | 11.57 | 0.17 |  | 5.32 | 0.93 | 5.94 | 0.86 |
| 47 | Lingual gyrus | Left | 14.22 | 0.29 | 11.03 | 0.25 |  | 3.47 | 0.97 | 3.30 | 0.95 |
| 48 | Lingual gyrus | Right | 14.34 | 0.25 | 11.15 | 0.29 |  | 3.27 | 0.97 | 3.15 | 0.96 |
| 49 | Superior occipital gyrus | Left | 13.56 | 0.35 | 11.66 | 0.18 |  | 4.58 | 0.95 | 4.94 | 0.90 |
| 50 | Superior occipital gyrus | Right | 13.30 | 0.38 | 12.16 | 0.11 |  | 4.78 | 0.95 | 5.28 | 0.89 |
| 51 | Middle occipital gyrus | Left | 12.45 | 0.45 | 11.77 | 0.14 |  | 5.13 | 0.94 | 5.76 | 0.86 |
| 52 | Middle occipital gyrus | Right | 12.57 | 0.46 | 12.16 | 0.08 |  | 5.51 | 0.93 | 6.73 | 0.81 |
| 53 | Inferior occipital gyrus | Left | 13.21 | 0.39 | 11.11 | 0.24 |  | 4.69 | 0.95 | 4.90 | 0.91 |
| 54 | Inferior occipital gyrus | Right | 13.94 | 0.31 | 11.13 | 0.23 |  | 3.75 | 0.97 | 3.46 | 0.95 |
| 55 | Fusiform gyrus | Left | 14.13 | 0.29 | 11.63 | 0.08 |  | 3.88 | 0.97 | 3.87 | 0.94 |
| 56 | Fusiform gyrus | Right | 13.76 | 0.35 | 11.52 | 0.13 |  | 4.17 | 0.96 | 4.26 | 0.92 |
| 57 | Postcentral gyrus | Left | 13.06 | 0.42 | 11.61 | 0.19 |  | 4.96 | 0.94 | 5.93 | 0.87 |
| 58 | Postcentral gyrus | Right | 13.67 | 0.36 | 11.25 | 0.26 |  | 4.64 | 0.95 | 4.65 | 0.92 |
| 59 | Superior parietal gyrus | Left | 13.58 | 0.37 | 11.57 | 0.21 |  | 4.53 | 0.95 | 4.89 | 0.90 |
| 60 | Superior parietal gyrus | Right | 14.07 | 0.33 | 11.11 | 0.25 |  | 4.12 | 0.96 | 4.05 | 0.94 |
| 61 | Inferior parietal, but supramarginal and angular gyri | Left | 12.31 | 0.49 | 10.92 | 0.32 |  | 5.64 | 0.93 | 6.59 | 0.84 |
| 62 | Inferior parietal, but supramarginal and angular gyri | Right | 12.99 | 0.43 | 11.07 | 0.28 |  | 5.06 | 0.94 | 5.68 | 0.88 |
| 63 | Supramarginal gyrus | Left | 12.73 | 0.46 | 12.18 | 0.10 |  | 5.32 | 0.93 | 7.37 | 0.80 |
| 64 | Supramarginal gyrus | Right | 13.05 | 0.43 | 11.24 | 0.23 |  | 5.23 | 0.94 | 5.16 | 0.89 |
| 65 | Angular gyrus | Left | 14.00 | 0.33 | 11.63 | 0.18 |  | 4.16 | 0.96 | 4.49 | 0.92 |
| 66 | Angular gyrus | Right | 13.42 | 0.40 | 11.38 | 0.23 |  | 4.70 | 0.95 | 4.59 | 0.91 |
| 67 | Precuneus | Left | 13.72 | 0.37 | 11.42 | 0.21 |  | 4.55 | 0.95 | 4.83 | 0.91 |
| 68 | Precuneus | Right | 12.62 | 0.50 | 11.37 | 0.21 |  | 5.53 | 0.93 | 5.47 | 0.86 |
| 69 | Paracentral lobule | Left | 14.63 | 0.22 | 10.71 | 0.43 |  | 3.37 | 0.97 | 3.10 | 0.96 |
| 70 | Paracentral lobule | Right | 15.17 | 0.06 | 11.57 | 0.21 |  | 1.71 | 0.99 | 1.17 | 0.99 |
| 71 | Caudate nucleus | Left | 12.24 | 0.51 | 11.37 | 0.39 |  | 5.37 | 0.93 | 5.87 | 0.87 |
| 72 | Caudate nucleus | Right | 11.88 | 0.53 | 10.80 | 0.50 |  | 5.50 | 0.92 | 5.35 | 0.89 |
| 73 | Lenticular nucleus putamen | Left | 14.16 | 0.29 | 11.80 | 0.19 |  | 3.91 | 0.96 | 4.34 | 0.92 |
| 74 | Lenticular nucleus putamen | Right | 13.70 | 0.37 | 11.47 | 0.25 |  | 4.56 | 0.95 | 4.86 | 0.90 |
| 75 | Lenticular nucleus, pallidum | Left | 13.86 | 0.33 | 11.65 | 0.16 |  | 4.21 | 0.96 | 4.12 | 0.93 |
| 76 | Lenticular nucleus, pallidum | Right | 14.11 | 0.31 | 12.29 | 0.05 |  | 3.87 | 0.96 | 4.05 | 0.92 |
| 77 | Thalamus | Left | 12.80 | 0.46 | 10.96 | 0.37 |  | 5.37 | 0.93 | 5.97 | 0.88 |
| 78 | Thalamus | Right | 12.84 | 0.44 | 10.56 | 0.43 |  | 5.41 | 0.93 | 5.34 | 0.90 |
| 79 | Heschl gyrus | Left | 14.49 | 0.25 | 12.36 | 0.04 |  | 3.30 | 0.97 | 3.47 | 0.95 |
| 80 | Heschl gyrus | Right | 13.08 | 0.42 | 10.79 | 0.36 |  | 4.86 | 0.94 | 5.57 | 0.90 |
| 81 | Superior temporal gyrus | Left | 11.87 | 0.55 | 10.22 | 0.49 |  | 5.41 | 0.93 | 5.47 | 0.89 |
| 82 | Superior temporal gyrus | Right | 11.71 | 0.54 | 9.70 | 0.50 |  | 5.47 | 0.92 | 5.42 | 0.88 |
| 83 | Temporal pole: superior temporal gyrus | Left | 12.49 | 0.48 | 10.35 | 0.45 |  | 5.47 | 0.93 | 4.82 | 0.91 |
| 84 | Temporal pole: superior temporal gyrus | Right | 11.97 | 0.52 | 11.28 | 0.39 |  | 5.71 | 0.92 | 5.84 | 0.87 |
| 85 | Middle temporal gyrus | Left | 11.07 | 0.61 | 10.65 | 0.38 |  | 6.05 | 0.91 | 7.49 | 0.80 |
| 86 | Middle temporal gyrus | Right | 12.67 | 0.47 | 10.78 | 0.27 |  | 5.08 | 0.94 | 5.49 | 0.87 |
| 87 | Temporal pole: middle temporal gyrus | Left | 13.30 | 0.41 | 11.17 | 0.37 |  | 4.35 | 0.95 | 4.20 | 0.92 |
| 88 | Temporal pole: middle temporal gyrus | Right | 13.44 | 0.39 | 11.24 | 0.23 |  | 4.39 | 0.95 | 4.55 | 0.91 |
| 89 | Inferior temporal gyrus | Left | 11.56 | 0.57 | 11.68 | 0.22 |  | 5.50 | 0.92 | 6.21 | 0.81 |
| 90 | Inferior temporal gyrus | Right | 12.05 | 0.53 | 11.42 | 0.24 |  | 5.25 | 0.93 | 5.67 | 0.85 |

Abbreviations: AAL, automated anatomical labeling; MAE, mean absolute error.

**Supplementary Table 3. Model performances of 48 models for fractional anisotropy map in training and test datasets.**

| **JHU-ICBM-Label number** | **White matter tracts** | **Left/Right** | **Before bias correction** | | | |  | **After bias correction** | | | |
| --- | --- | --- | --- | --- | --- | --- | --- | --- | --- | --- | --- |
|  |  |  | **Training dataset** | | **Test dataset** | |  | **Training dataset** | | **Test dataset** | |
|  |  |  | **MAE (years)** | **correlation coefficient** | **MAE (years)** | **correlation coefficient** |  | **MAE (years)** | **correlation coefficient** | **MAE (years)** | **correlation coefficient** |
| 1 | Middle cerebellar peduncle | – | 7.90 | 0.81 | 8.14 | 0.76 |  | 6.12 | 0.91 | 7.08 | 0.87 |
| 2 | Pontine crossing tract | – | 13.14 | 0.41 | 11.64 | 0.44 |  | 4.84 | 0.94 | 4.72 | 0.93 |
| 3 | Genu of corpus callosum | – | 9.31 | 0.72 | 9.54 | 0.64 |  | 6.40 | 0.90 | 6.71 | 0.86 |
| 4 | Body of corpus callosum | – | 11.16 | 0.62 | 10.30 | 0.65 |  | 6.28 | 0.90 | 6.65 | 0.89 |
| 5 | Splenium of corpus callosum | – | 10.65 | 0.65 | 10.07 | 0.61 |  | 6.29 | 0.90 | 6.65 | 0.87 |
| 6 | Fornix (fornix column and body of fornix) | – | 11.14 | 0.60 | 10.88 | 0.53 |  | 6.24 | 0.91 | 7.51 | 0.85 |
| 7 | Corticospinal tract | Right | 9.00 | 0.76 | 8.42 | 0.76 |  | 6.65 | 0.90 | 8.07 | 0.88 |
| 8 | Corticospinal tract | Left | 8.94 | 0.75 | 7.79 | 0.78 |  | 6.66 | 0.90 | 7.22 | 0.89 |
| 9 | Medial lemniscus | Right | 12.15 | 0.50 | 11.60 | 0.32 |  | 5.87 | 0.92 | 6.74 | 0.83 |
| 10 | Medial lemniscus | Left | 11.64 | 0.55 | 11.14 | 0.41 |  | 5.84 | 0.91 | 7.61 | 0.83 |
| 11 | Inferior cerebellar peduncle | Right | 9.03 | 0.76 | 8.09 | 0.77 |  | 6.56 | 0.90 | 7.21 | 0.89 |
| 12 | Inferior cerebellar peduncle | Left | 8.50 | 0.78 | 7.77 | 0.76 |  | 6.34 | 0.90 | 7.64 | 0.87 |
| 13 | Superior cerebellar peduncle | Right | 9.88 | 0.69 | 9.41 | 0.62 |  | 6.48 | 0.90 | 7.57 | 0.83 |
| 14 | Superior cerebellar peduncle | Left | 9.02 | 0.74 | 10.43 | 0.58 |  | 6.38 | 0.90 | 8.55 | 0.79 |
| 15 | Cerebral peduncle | Right | 9.80 | 0.69 | 9.03 | 0.63 |  | 6.39 | 0.90 | 6.61 | 0.85 |
| 16 | Cerebral peduncle | Left | 10.46 | 0.64 | 9.36 | 0.63 |  | 6.45 | 0.90 | 6.55 | 0.87 |
| 17 | Anterior limb of internal capsule | Right | 9.44 | 0.72 | 10.24 | 0.65 |  | 6.33 | 0.90 | 7.34 | 0.85 |
| 18 | Anterior limb of internal capsule | Left | 9.83 | 0.70 | 8.67 | 0.64 |  | 6.36 | 0.90 | 5.69 | 0.87 |
| 19 | Posterior limb of internal capsule | Right | 10.62 | 0.65 | 9.30 | 0.63 |  | 6.32 | 0.90 | 6.33 | 0.88 |
| 20 | Posterior limb of internal capsule | Left | 10.48 | 0.66 | 9.69 | 0.63 |  | 6.45 | 0.90 | 6.61 | 0.87 |
| 21 | Retrolenticular part of internal capsule | Right | 9.78 | 0.69 | 10.71 | 0.56 |  | 6.24 | 0.90 | 7.32 | 0.83 |
| 22 | Retrolenticular part of internal capsule | Left | 9.47 | 0.71 | 10.01 | 0.59 |  | 6.42 | 0.90 | 7.37 | 0.83 |
| 23 | Anterior corona radiata | Right | 11.01 | 0.62 | 10.46 | 0.60 |  | 6.40 | 0.90 | 6.91 | 0.87 |
| 24 | Anterior corona radiata | Left | 11.12 | 0.62 | 10.10 | 0.62 |  | 6.36 | 0.90 | 5.99 | 0.89 |
| 25 | Superior corona radiata | Right | 10.96 | 0.63 | 9.22 | 0.65 |  | 6.18 | 0.91 | 5.79 | 0.90 |
| 26 | Superior corona radiata | Left | 11.14 | 0.62 | 10.00 | 0.65 |  | 6.14 | 0.91 | 6.44 | 0.89 |
| 27 | Posterior corona radiata | Right | 11.75 | 0.57 | 10.94 | 0.56 |  | 6.06 | 0.91 | 6.49 | 0.88 |
| 28 | Posterior corona radiata | Left | 11.27 | 0.61 | 10.43 | 0.62 |  | 6.28 | 0.91 | 6.18 | 0.90 |
| 29 | Posterior thalamic radiation | Right | 11.93 | 0.55 | 10.23 | 0.51 |  | 5.92 | 0.91 | 6.28 | 0.87 |
| 30 | Posterior thalamic radiation | Left | 11.26 | 0.61 | 10.05 | 0.56 |  | 6.38 | 0.90 | 5.79 | 0.87 |
| 31 | Sagittal stratum | Right | 11.98 | 0.54 | 10.97 | 0.46 |  | 5.82 | 0.92 | 6.04 | 0.87 |
| 32 | Sagittal stratum | Left | 11.15 | 0.62 | 10.62 | 0.51 |  | 6.36 | 0.90 | 6.73 | 0.84 |
| 33 | External capsule | Right | 8.09 | 0.79 | 8.13 | 0.70 |  | 6.27 | 0.90 | 6.54 | 0.85 |
| 34 | External capsule | Left | 8.14 | 0.80 | 7.43 | 0.76 |  | 6.16 | 0.91 | 5.89 | 0.88 |
| 35 | Cingulum (cingulate gyrus) | Right | 11.52 | 0.60 | 9.78 | 0.55 |  | 6.10 | 0.91 | 6.05 | 0.87 |
| 36 | Cingulum (cingulate gyrus) | Left | 11.90 | 0.55 | 11.25 | 0.40 |  | 5.93 | 0.91 | 5.45 | 0.87 |
| 37 | Cingulum (hippocampus) | Right | 10.43 | 0.66 | 9.91 | 0.49 |  | 6.22 | 0.90 | 6.57 | 0.82 |
| 38 | Cingulum (hippocampus) | Left | 11.23 | 0.60 | 10.16 | 0.49 |  | 6.35 | 0.91 | 7.22 | 0.84 |
| 39 | Fornix (cres/stria terminalis) | Right | 10.90 | 0.64 | 10.22 | 0.64 |  | 6.34 | 0.90 | 7.05 | 0.87 |
| 40 | Fornix (cres/stria terminalis) | Left | 11.00 | 0.63 | 9.84 | 0.59 |  | 6.30 | 0.90 | 7.12 | 0.85 |
| 41 | Superior longitudinal fasciculus | Right | 10.79 | 0.63 | 10.70 | 0.59 |  | 6.28 | 0.90 | 6.37 | 0.88 |
| 42 | Superior longitudinal fasciculus | Left | 10.76 | 0.64 | 10.98 | 0.50 |  | 6.24 | 0.91 | 6.75 | 0.83 |
| 43 | Superior fronto-occipital fasciculus | Right | 11.41 | 0.60 | 10.17 | 0.56 |  | 6.27 | 0.90 | 6.17 | 0.87 |
| 44 | Superior fronto-occipital fasciculus | Left | 11.44 | 0.58 | 10.55 | 0.54 |  | 6.15 | 0.91 | 6.33 | 0.87 |
| 45 | Uncinate fasciculus | Right | 11.41 | 0.59 | 10.92 | 0.48 |  | 6.06 | 0.91 | 6.18 | 0.86 |
| 46 | Uncinate fasciculus | Left | 13.61 | 0.41 | 11.32 | 0.31 |  | 4.94 | 0.94 | 5.21 | 0.90 |
| 47 | Tapetum | Right | 12.23 | 0.54 | 11.40 | 0.53 |  | 5.85 | 0.91 | 6.45 | 0.89 |
| 48 | Tapetum | Left | 11.77 | 0.56 | 10.98 | 0.53 |  | 6.08 | 0.91 | 6.73 | 0.88 |

Abbreviations: MAE, mean absolute error.

**Supplementary Table 4. Brain age gap differences between individuals with BD and MDD compared to age- and sex-matched healthy controls in 90 models for gray matter map.**

| **AAL label number** | **Regions** | **Left/Right** | **Healthy controls**  **(*n* = 110)** | | **BD group**  **(*n* = 110)** | | ***F*** | **adjusted *p*** | ***partial η^2^*** |  | **Healthy controls**  **(*n* = 68)** | | **MDD group**  **(*n* = 68)** | | ***F*** | **adjusted *p*** | ***partial η^2^*** |
| --- | --- | --- | --- | --- | --- | --- | --- | --- | --- | --- | --- | --- | --- | --- | --- | --- | --- |
|  |  |  | **Mean (years)** | **SD** | **Mean (years)** | **SD** |  |  |  |  | **Mean (years)** | **SD** | **Mean (years)** | **SD** |  |  |  |
| 1 | Precental gyrus | Left | -0.67 | 6.84 | 3.32 | 9.84 | 10.57 | **0.004 *** | 0.047 |  | -0.42 | 6.19 | 2.13 | 9.54 | 3.17 | 0.093 | 0.024 |
| 2 | Precental gyrus | Right | -0.73 | 7.92 | 3.63 | 9.56 | 10.05 | **0.005 *** | 0.045 |  | -2.24 | 6.46 | 2.57 | 9.39 | 11.07 | **0.005 *** | 0.079 |
| 3 | Superior frontal gyrus, dorsolateral | Left | -1.74 | 8.12 | 3.79 | 10.55 | 11.68 | **0.003 *** | 0.052 |  | -1.97 | 7.30 | 3.16 | 9.51 | 12.30 | **0.003 *** | 0.086 |
| 4 | Superior frontal gyrus, dorsolateral | Right | -1.98 | 9.15 | 4.33 | 11.01 | 14.06 | **0.001 *** | 0.062 |  | -2.37 | 8.09 | 2.34 | 10.37 | 8.04 | **0.012 *** | 0.058 |
| 5 | Superior frontal gyrus, orbital part | Left | -1.43 | 7.84 | 1.51 | 8.54 | 5.35 | **0.033 *** | 0.024 |  | -2.92 | 7.77 | 2.05 | 8.36 | 15.96 | **0.001 *** | 0.109 |
| 6 | Superior frontal gyrus, orbital part | Right | -1.95 | 8.87 | 2.48 | 8.72 | 9.78 | **0.006 *** | 0.044 |  | -3.11 | 8.50 | 2.85 | 10.60 | 11.82 | **0.004 *** | 0.083 |
| 7 | Middle frontal gyrus | Left | -1.00 | 8.90 | 3.15 | 10.01 | 3.72 | 0.072 | 0.017 |  | -1.61 | 8.59 | 3.04 | 9.85 | 6.90 | **0.018 *** | 0.050 |
| 8 | Middle frontal gyrus | Right | -0.22 | 8.71 | 4.31 | 10.83 | 6.25 | **0.023 *** | 0.028 |  | -0.40 | 8.16 | 4.35 | 9.78 | 7.59 | **0.014 *** | 0.055 |
| 9 | Middle frontal gyrus, orbital part | Left | 0.16 | 7.42 | 3.42 | 8.94 | 5.00 | **0.037 *** | 0.023 |  | -0.49 | 7.54 | 3.68 | 7.96 | 10.56 | **0.005 *** | 0.075 |
| 10 | Middle frontal gyrus, orbital part | Right | -1.64 | 8.59 | 2.00 | 9.90 | 3.83 | 0.068 | 0.018 |  | -2.05 | 8.06 | 2.46 | 9.79 | 7.24 | **0.017 *** | 0.053 |
| 11 | Inferior frontal gyrus, opercular part | Left | -0.82 | 8.23 | 2.94 | 9.98 | 6.87 | **0.018 *** | 0.031 |  | -1.07 | 8.34 | 3.66 | 8.24 | 8.79 | **0.010 *** | 0.063 |
| 12 | Inferior frontal gyrus, opercular part | Right | -1.68 | 8.52 | 2.09 | 9.42 | 11.82 | **0.003 *** | 0.052 |  | -2.08 | 8.58 | 2.48 | 10.20 | 8.24 | **0.012 *** | 0.060 |
| 13 | Inferior frontal gyrus, triangular part | Left | 0.52 | 8.93 | 4.50 | 9.41 | 5.47 | **0.032 *** | 0.025 |  | 0.38 | 9.13 | 3.90 | 9.04 | 5.33 | **0.033 *** | 0.039 |
| 14 | Inferior frontal gyrus, triangular part | Right | -0.25 | 9.88 | 2.60 | 9.34 | 3.30 | 0.087 | 0.015 |  | -0.93 | 9.37 | 2.50 | 9.33 | 4.87 | **0.040 *** | 0.036 |
| 15 | Inferior frontal gyrus, orbital part | Left | -0.51 | 8.93 | 5.40 | 10.36 | 11.09 | **0.004 *** | 0.049 |  | -1.70 | 9.22 | 3.62 | 9.36 | 7.63 | **0.014 *** | 0.055 |
| 16 | Inferior frontal gyrus, orbital part | Right | -0.01 | 8.43 | 4.08 | 10.03 | 7.05 | **0.018 *** | 0.032 |  | -0.95 | 8.43 | 3.97 | 10.09 | 8.79 | **0.010 *** | 0.063 |
| 17 | Rolandic operculum | Left | 0.76 | 7.22 | 4.46 | 9.94 | 5.27 | **0.033 *** | 0.024 |  | -0.16 | 6.94 | 3.35 | 9.50 | 4.90 | **0.040 *** | 0.036 |
| 18 | Rolandic operculum | Right | -1.48 | 7.78 | 3.58 | 10.59 | 12.50 | **0.003 *** | 0.055 |  | -2.48 | 7.40 | 3.05 | 9.71 | 14.92 | **0.001 *** | 0.103 |
| 19 | Supplementary motor area | Left | -1.79 | 8.10 | 4.60 | 10.35 | 21.56 | **< 0.001 *** | 0.092 |  | -2.37 | 7.95 | 3.00 | 8.68 | 12.29 | **0.003 *** | 0.086 |
| 20 | Supplementary motor area | Right | 0.65 | 8.53 | 5.32 | 10.75 | 8.24 | **0.011 *** | 0.037 |  | 0.01 | 9.02 | 3.08 | 9.52 | 3.18 | 0.093 | 0.024 |
| 21 | Olfactory cortex | Left | -1.56 | 7.80 | 4.84 | 9.20 | 20.95 | **< 0.001 *** | 0.089 |  | -2.45 | 8.10 | 5.51 | 10.40 | 24.68 | **< 0.001 *** | 0.160 |
| 22 | Olfactory cortex | Right | 0.37 | 7.95 | 4.31 | 9.06 | 8.64 | **0.009 *** | 0.039 |  | 0.32 | 8.06 | 5.81 | 9.54 | 10.61 | **0.005 *** | 0.075 |
| 23 | Superior frontal gyrus, medial | Left | -1.65 | 8.67 | 5.53 | 10.24 | 19.61 | **< 0.001 *** | 0.084 |  | -2.87 | 8.70 | 4.68 | 10.10 | 17.52 | **0.001 *** | 0.119 |
| 24 | Superior frontal gyrus, medial | Right | -0.72 | 8.56 | 3.63 | 9.58 | 7.77 | **0.013 *** | 0.035 |  | -1.81 | 9.01 | 4.17 | 10.14 | 11.12 | **0.005 *** | 0.079 |
| 25 | Superior frontal gyrus, medial orbital | Left | -1.84 | 9.56 | 5.49 | 9.76 | 20.20 | **< 0.001 *** | 0.086 |  | -3.06 | 9.35 | 6.63 | 10.27 | 29.22 | **< 0.001 *** | 0.184 |
| 26 | Superior frontal gyrus, medial orbital | Right | -1.35 | 8.38 | 4.00 | 8.80 | 12.77 | **0.002 *** | 0.056 |  | -2.36 | 7.68 | 4.75 | 9.79 | 21.24 | **< 0.001 *** | 0.140 |
| 27 | Gyrus rectus | Left | -0.85 | 8.28 | 3.06 | 9.13 | 5.40 | **0.032 *** | 0.025 |  | -1.44 | 8.10 | 5.79 | 9.31 | 21.27 | **< 0.001 *** | 0.141 |
| 28 | Gyrus rectus | Right | -2.33 | 8.95 | 5.06 | 10.21 | 23.60 | **< 0.001 *** | 0.099 |  | -2.85 | 8.77 | 5.75 | 10.19 | 25.04 | **< 0.001 *** | 0.162 |
| 29 | Insula | Left | -0.45 | 7.47 | 5.59 | 9.17 | 15.17 | **0.001 *** | 0.066 |  | -0.90 | 7.69 | 5.05 | 9.39 | 13.99 | **0.002 *** | 0.097 |
| 30 | Insula | Right | -0.08 | 7.77 | 5.34 | 10.26 | 11.13 | **0.004 *** | 0.049 |  | -0.82 | 8.09 | 4.83 | 8.92 | 12.33 | **0.003 *** | 0.087 |
| 31 | Anterior cingulate and paracingulate gyri | Left | 0.15 | 8.79 | 4.53 | 9.63 | 5.93 | **0.026 *** | 0.027 |  | -0.87 | 8.84 | 5.80 | 10.15 | 14.10 | **0.002 *** | 0.098 |
| 32 | Anterior cingulate and paracingulate gyri | Right | 0.11 | 7.61 | 3.14 | 8.93 | 2.50 | 0.136 | 0.012 |  | -0.61 | 7.67 | 4.54 | 8.66 | 7.36 | **0.016 *** | 0.054 |
| 33 | Median cingulate and paracingulate gyri | Left | -0.42 | 7.51 | 2.58 | 9.78 | 4.67 | **0.044 *** | 0.021 |  | -1.86 | 7.11 | 3.27 | 9.85 | 10.20 | **0.006 *** | 0.073 |
| 34 | Median cingulate and paracingulate gyri | Right | -0.14 | 6.60 | 3.82 | 9.29 | 9.85 | **0.006 *** | 0.044 |  | -0.43 | 6.36 | 3.18 | 8.99 | 4.57 | **0.046 *** | 0.034 |
| 35 | Posterior cingulate gyrus | Left | -0.72 | 7.26 | 2.18 | 7.71 | 5.50 | **0.032 *** | 0.025 |  | -1.52 | 6.46 | 0.46 | 7.90 | 5.38 | **0.033 *** | 0.040 |
| 36 | Posterior cingulate gyrus | Right | 0.68 | 5.20 | 0.86 | 5.73 | 0.03 | 0.855 | 0.000 |  | 0.23 | 4.91 | 1.59 | 5.81 | 2.44 | 0.138 | 0.018 |
| 37 | Hippocampus | Left | -1.34 | 7.64 | 3.31 | 7.46 | 13.40 | **0.002 *** | 0.059 |  | -1.74 | 7.92 | 3.21 | 8.33 | 8.18 | **0.012 *** | 0.059 |
| 38 | Hippocampus | Right | -1.43 | 8.89 | 2.92 | 9.23 | 6.38 | **0.022 *** | 0.029 |  | -2.19 | 8.60 | 2.47 | 10.28 | 6.93 | **0.018 *** | 0.051 |
| 39 | Parahippocampal gyrus | Left | -0.65 | 8.04 | 3.26 | 9.89 | 5.12 | **0.035 *** | 0.023 |  | -1.42 | 7.82 | 4.00 | 10.53 | 8.37 | **0.012 *** | 0.060 |
| 40 | Parahippocampal gyrus | Right | 0.69 | 7.95 | 2.41 | 10.20 | 0.17 | 0.686 | 0.001 |  | 0.25 | 7.94 | 2.76 | 9.25 | 2.10 | 0.167 | 0.016 |
| 41 | Amygdala | Left | -2.05 | 8.82 | 3.32 | 9.45 | 14.55 | **0.001 *** | 0.064 |  | -1.81 | 8.69 | 3.40 | 8.85 | 10.07 | **0.006 *** | 0.072 |
| 42 | Amygdala | Right | 0.16 | 8.61 | 2.02 | 8.83 | 1.94 | 0.186 | 0.009 |  | -0.51 | 8.70 | 3.43 | 10.33 | 4.76 | **0.042 *** | 0.035 |
| 43 | Calcarine fissure and surrounding cortex | Left | -0.13 | 8.26 | 3.28 | 9.14 | 3.46 | 0.081 | 0.016 |  | -0.56 | 7.65 | 1.94 | 9.07 | 4.23 | 0.054 | 0.032 |
| 44 | Calcarine fissure and surrounding cortex | Right | -0.29 | 7.66 | 1.66 | 8.68 | 0.40 | 0.547 | 0.002 |  | -1.39 | 7.00 | 2.46 | 8.80 | 4.86 | **0.040 *** | 0.036 |
| 45 | Cuneus | Left | -0.11 | 8.56 | 2.94 | 9.20 | 2.35 | 0.148 | 0.011 |  | -0.85 | 7.90 | 1.66 | 9.07 | 3.11 | 0.095 | 0.023 |
| 46 | Cuneus | Right | 0.05 | 8.21 | 2.73 | 8.63 | 2.16 | 0.163 | 0.010 |  | -0.45 | 8.09 | 2.19 | 8.23 | 4.08 | 0.058 | 0.030 |
| 47 | Lingual gyrus | Left | -0.82 | 8.08 | 3.31 | 8.90 | 6.00 | **0.025 *** | 0.027 |  | -1.20 | 8.65 | 2.96 | 9.03 | 3.55 | 0.077 | 0.027 |
| 48 | Lingual gyrus | Right | -0.34 | 8.56 | 3.10 | 9.13 | 2.85 | 0.112 | 0.013 |  | -0.42 | 9.25 | 2.70 | 8.87 | 2.76 | 0.114 | 0.021 |
| 49 | Superior occipital gyrus | Left | -1.70 | 8.27 | 1.33 | 8.55 | 2.29 | 0.152 | 0.011 |  | -2.16 | 7.64 | 2.11 | 8.37 | 7.71 | **0.014 *** | 0.056 |
| 50 | Superior occipital gyrus | Right | -0.81 | 7.66 | 3.13 | 9.08 | 8.87 | **0.009 *** | 0.040 |  | -0.70 | 7.81 | 1.25 | 7.82 | 1.48 | 0.240 | 0.011 |
| 51 | Middle occipital gyrus | Left | 0.14 | 8.18 | 4.22 | 9.93 | 6.92 | **0.018 *** | 0.031 |  | -0.29 | 8.28 | 2.85 | 10.04 | 1.92 | 0.185 | 0.015 |
| 52 | Middle occipital gyrus | Right | -0.23 | 7.85 | 4.36 | 9.12 | 8.16 | **0.011 *** | 0.037 |  | -0.61 | 7.56 | 3.34 | 8.97 | 5.04 | **0.038 *** | 0.037 |
| 53 | Inferior occipital gyrus | Left | -0.26 | 9.41 | 3.74 | 8.45 | 5.57 | **0.031 *** | 0.025 |  | -0.72 | 10.11 | 2.82 | 8.59 | 1.24 | 0.279 | 0.009 |
| 54 | Inferior occipital gyrus | Right | 0.39 | 8.86 | 4.58 | 8.13 | 10.01 | **0.005 *** | 0.045 |  | -0.46 | 8.72 | 3.19 | 8.78 | 5.36 | **0.033 *** | 0.040 |
| 55 | Fusiform gyrus | Left | -0.84 | 9.09 | 3.34 | 7.86 | 7.01 | **0.018 *** | 0.032 |  | -1.28 | 9.22 | 4.14 | 9.40 | 6.93 | **0.018 *** | 0.051 |
| 56 | Fusiform gyrus | Right | -0.04 | 8.81 | 2.47 | 7.53 | 0.89 | 0.373 | 0.004 |  | 0.20 | 9.00 | 3.67 | 9.22 | 3.70 | 0.072 | 0.028 |
| 57 | Postcentral gyrus | Left | -0.68 | 8.06 | 2.64 | 8.95 | 4.07 | 0.060 | 0.019 |  | -1.72 | 7.66 | 1.47 | 9.39 | 3.00 | 0.100 | 0.023 |
| 58 | Postcentral gyrus | Right | -0.87 | 7.97 | 3.11 | 9.52 | 7.13 | **0.017 *** | 0.032 |  | -2.05 | 7.40 | 2.12 | 10.32 | 5.93 | **0.027 *** | 0.044 |
| 59 | Superior parietal gyrus | Left | -2.00 | 9.23 | 2.81 | 9.43 | 7.38 | **0.016 *** | 0.033 |  | -2.85 | 8.58 | 1.81 | 8.65 | 8.69 | **0.011 *** | 0.063 |
| 60 | Superior parietal gyrus | Right | -1.05 | 8.41 | 4.06 | 9.36 | 11.90 | **0.003 *** | 0.053 |  | -2.01 | 7.97 | 2.26 | 9.21 | 8.13 | **0.012 *** | 0.059 |
| 61 | Inferior parietal, but supramarginal and angular gyri | Left | -1.08 | 9.06 | 3.65 | 8.42 | 11.58 | **0.003 *** | 0.051 |  | -2.16 | 8.51 | 1.29 | 10.24 | 5.50 | **0.032 *** | 0.041 |
| 62 | Inferior parietal, but supramarginal and angular gyri | Right | -0.85 | 10.49 | 2.84 | 9.84 | 3.53 | 0.079 | 0.016 |  | -1.93 | 10.76 | 1.49 | 10.88 | 4.30 | 0.053 | 0.032 |
| 63 | Supramarginal gyrus | Left | -0.89 | 9.04 | 1.85 | 9.59 | 1.64 | 0.224 | 0.008 |  | -1.14 | 9.31 | 1.45 | 10.74 | 2.33 | 0.145 | 0.018 |
| 64 | Supramarginal gyrus | Right | -0.24 | 8.59 | 3.39 | 9.75 | 6.48 | **0.021 *** | 0.029 |  | -0.47 | 8.35 | 2.72 | 10.30 | 5.77 | **0.028 *** | 0.043 |
| 65 | Angular gyrus | Left | -0.36 | 8.63 | 4.40 | 8.75 | 11.33 | **0.003 *** | 0.050 |  | -0.87 | 9.08 | 3.59 | 10.01 | 6.27 | **0.024 *** | 0.046 |
| 66 | Angular gyrus | Right | 0.05 | 8.76 | 2.41 | 10.63 | 0.66 | 0.441 | 0.003 |  | -0.44 | 8.43 | 3.13 | 11.58 | 6.51 | **0.021 *** | 0.048 |
| 67 | Precuneus | Left | -0.85 | 8.71 | 3.02 | 10.38 | 4.47 | **0.049 *** | 0.020 |  | -1.62 | 8.13 | 1.64 | 9.51 | 3.26 | 0.091 | 0.024 |
| 68 | Precuneus | Right | 0.08 | 8.76 | 3.14 | 9.64 | 3.46 | 0.081 | 0.016 |  | -0.70 | 8.34 | 1.14 | 9.31 | 1.01 | 0.328 | 0.008 |
| 69 | Paracentral lobule | Left | -1.50 | 8.46 | 2.92 | 9.86 | 9.22 | **0.007 *** | 0.041 |  | -2.90 | 8.16 | 1.83 | 9.71 | 10.23 | **0.006 *** | 0.073 |
| 70 | Paracentral lobule | Right | -0.39 | 8.27 | 3.72 | 9.22 | 6.82 | **0.018 *** | 0.031 |  | -1.05 | 7.34 | 2.96 | 8.89 | 8.59 | **0.011 *** | 0.062 |
| 71 | Caudate nucleus | Left | -0.57 | 7.36 | 5.53 | 9.11 | 18.11 | **< 0.001 *** | 0.078 |  | -1.07 | 6.31 | 3.80 | 9.71 | 10.08 | **0.006 *** | 0.072 |
| 72 | Caudate nucleus | Right | -0.77 | 7.88 | 6.54 | 8.67 | 30.20 | **< 0.001 *** | 0.124 |  | -0.94 | 7.47 | 3.85 | 8.95 | 13.12 | **0.003 *** | 0.092 |
| 73 | Lenticular nucleus putamen | Left | 0.75 | 7.13 | 2.97 | 8.47 | 1.59 | 0.229 | 0.007 |  | 1.57 | 6.50 | 1.40 | 7.22 | 0.51 | 0.483 | 0.004 |
| 74 | Lenticular nucleus putamen | Right | 0.59 | 7.78 | 3.37 | 7.72 | 6.13 | **0.024 *** | 0.028 |  | 1.17 | 8.20 | 3.34 | 6.95 | 1.50 | 0.238 | 0.011 |
| 75 | Lenticular nucleus, pallidum | Left | 0.01 | 7.84 | 3.06 | 7.27 | 6.51 | **0.021 *** | 0.030 |  | -0.57 | 8.17 | 1.22 | 7.99 | 0.61 | 0.446 | 0.005 |
| 76 | Lenticular nucleus, pallidum | Right | 1.74 | 8.42 | 2.48 | 8.54 | 1.01 | 0.344 | 0.005 |  | 1.39 | 9.00 | 0.29 | 9.12 | 0.12 | 0.731 | 0.001 |
| 77 | Thalamus | Left | -0.66 | 6.61 | 4.31 | 7.65 | 25.46 | **< 0.001 *** | 0.106 |  | -1.19 | 6.84 | 2.64 | 7.72 | 6.17 | **0.024 *** | 0.045 |
| 78 | Thalamus | Right | -1.61 | 6.33 | 3.37 | 8.17 | 21.48 | **< 0.001 *** | 0.091 |  | -2.63 | 5.61 | 2.36 | 8.06 | 18.24 | **< 0.001 *** | 0.123 |
| 79 | Heschl gyrus | Left | -0.51 | 7.71 | 5.72 | 9.38 | 16.89 | **0.001 *** | 0.073 |  | -1.68 | 7.94 | 3.34 | 8.68 | 11.28 | **0.005 *** | 0.080 |
| 80 | Heschl gyrus | Right | -1.13 | 8.30 | 3.59 | 8.83 | 8.01 | **0.012 *** | 0.036 |  | -2.40 | 8.39 | 4.76 | 9.05 | 19.10 | **< 0.001 *** | 0.128 |
| 81 | Superior temporal gyrus | Left | -0.55 | 7.80 | 4.84 | 8.67 | 11.86 | **0.003 *** | 0.052 |  | -2.22 | 7.61 | 4.62 | 8.02 | 25.56 | **< 0.001 *** | 0.164 |
| 82 | Superior temporal gyrus | Right | -0.18 | 8.32 | 3.59 | 10.13 | 5.14 | **0.035 *** | 0.023 |  | -1.01 | 7.90 | 3.02 | 9.14 | 6.73 | **0.020 *** | 0.049 |
| 83 | Temporal pole: superior temporal gyrus | Left | -1.24 | 8.47 | 4.93 | 9.90 | 15.86 | **0.001 *** | 0.069 |  | -2.88 | 8.37 | 4.09 | 9.88 | 17.00 | **0.001 *** | 0.116 |
| 84 | Temporal pole: superior temporal gyrus | Right | -1.62 | 9.04 | 2.95 | 8.99 | 11.62 | **0.003 *** | 0.052 |  | -2.74 | 8.67 | 2.74 | 9.52 | 10.62 | **0.005 *** | 0.076 |
| 85 | Middle temporal gyrus | Left | -1.59 | 8.31 | 3.16 | 9.52 | 8.80 | **0.009 *** | 0.040 |  | -2.27 | 8.62 | 1.71 | 9.49 | 5.40 | **0.033 *** | 0.040 |
| 86 | Middle temporal gyrus | Right | -1.27 | 9.29 | 4.20 | 10.08 | 10.92 | **0.004 *** | 0.049 |  | -2.02 | 9.17 | 3.17 | 10.05 | 8.21 | **0.012 *** | 0.059 |
| 87 | Temporal pole: middle temporal gyrus | Left | -0.22 | 9.06 | 1.51 | 8.36 | 0.28 | 0.608 | 0.001 |  | -1.08 | 8.59 | 1.60 | 8.94 | 1.82 | 0.195 | 0.014 |
| 88 | Temporal pole: middle temporal gyrus | Right | -0.62 | 9.28 | 0.90 | 9.75 | 0.42 | 0.539 | 0.002 |  | -1.53 | 8.99 | 2.60 | 10.15 | 6.06 | **0.025 *** | 0.045 |
| 89 | Inferior temporal gyrus | Left | -0.26 | 8.34 | 3.14 | 8.64 | 3.04 | 0.100 | 0.014 |  | -1.33 | 8.56 | 3.01 | 9.07 | 6.63 | **0.021 *** | 0.048 |
| 90 | Inferior temporal gyrus | Right | -0.14 | 9.05 | 4.19 | 9.48 | 6.54 | **0.021 *** | 0.030 |  | -1.50 | 9.04 | 2.91 | 10.47 | 6.20 | **0.024 *** | 0.046 |

Abbreviations: AAL, automated anatomical labeling; BD, bipolar disorder; FDR, false discovery rate; MDD, major depressive disorder; SD, standard deviation.

Significant differences after FDR correction are indicated with * (adjusted *p* < 0.05).

**Supplementary Table 5. Brain age gap differences between individuals with BD and MDD compared to age- and sex-matched healthy controls in 90 models for standard deviation map.**

| **AAL label number** | **Regions** | **Left/Right** | **Healthy controls**  **(*n* = 110)** | | **BD group**  **(*n* = 110)** | | ***F*** | **adjusted *p*** | ***partial η^2^*** |  | **Healthy controls**  **(*n* = 68)** | | **MDD group**  **(*n* = 68)** | | ***F*** | **adjusted *p*** | ***partial η^2^*** |
| --- | --- | --- | --- | --- | --- | --- | --- | --- | --- | --- | --- | --- | --- | --- | --- | --- | --- |
|  |  |  | **Mean (years)** | **SD** | **Mean (years)** | **SD** |  |  |  |  | **Mean (years)** | **SD** | **Mean (years)** | **SD** |  |  |  |
| 1 | Precental gyrus | Left | -0.47 | 6.19 | -0.34 | 7.09 | 0.11 | 0.931 | 0.001 |  | 0.40 | 6.42 | 1.20 | 6.46 | 0.60 | 0.774 | 0.005 |
| 2 | Precental gyrus | Right | 0.08 | 5.76 | -0.07 | 7.07 | 0.03 | 0.976 | 0.000 |  | 1.21 | 5.87 | 2.99 | 6.11 | 2.76 | 0.500 | 0.021 |
| 3 | Superior frontal gyrus, dorsolateral | Left | 0.15 | 5.17 | 0.94 | 6.23 | 0.55 | 0.692 | 0.003 |  | 0.46 | 5.15 | 1.79 | 5.84 | 1.36 | 0.623 | 0.010 |
| 4 | Superior frontal gyrus, dorsolateral | Right | -0.44 | 5.80 | 1.10 | 7.29 | 1.24 | 0.560 | 0.006 |  | 0.52 | 6.06 | 2.12 | 6.46 | 0.99 | 0.659 | 0.008 |
| 5 | Superior frontal gyrus, orbital part | Left | -0.64 | 6.36 | 0.70 | 6.27 | 0.41 | 0.755 | 0.002 |  | -1.00 | 6.40 | 1.04 | 5.76 | 1.42 | 0.623 | 0.011 |
| 6 | Superior frontal gyrus, orbital part | Right | 0.65 | 6.30 | 1.28 | 5.35 | 0.00 | 0.989 | 0.000 |  | 0.28 | 6.65 | 1.02 | 5.22 | 0.14 | 0.841 | 0.001 |
| 7 | Middle frontal gyrus | Left | -0.81 | 5.71 | 0.22 | 6.75 | 1.06 | 0.597 | 0.005 |  | -0.33 | 5.61 | 1.09 | 6.23 | 1.34 | 0.623 | 0.010 |
| 8 | Middle frontal gyrus | Right | -0.20 | 6.97 | 0.64 | 8.28 | 0.40 | 0.755 | 0.002 |  | 0.76 | 7.56 | 1.17 | 6.48 | 0.18 | 0.832 | 0.001 |
| 9 | Middle frontal gyrus, orbital part | Left | -0.80 | 3.72 | -0.64 | 4.31 | 0.72 | 0.669 | 0.003 |  | -0.09 | 4.19 | 0.35 | 5.17 | 0.09 | 0.841 | 0.001 |
| 10 | Middle frontal gyrus, orbital part | Right | 0.68 | 4.33 | -0.38 | 4.72 | 5.11 | 0.089 | 0.023 |  | 1.35 | 4.58 | 0.33 | 4.36 | 3.24 | 0.418 | 0.024 |
| 11 | Inferior frontal gyrus, opercular part | Left | -0.54 | 7.87 | 0.67 | 8.70 | 0.23 | 0.862 | 0.001 |  | 0.53 | 8.44 | 1.25 | 8.18 | 0.10 | 0.841 | 0.001 |
| 12 | Inferior frontal gyrus, opercular part | Right | -0.63 | 6.23 | 0.16 | 7.51 | 0.62 | 0.692 | 0.003 |  | 0.59 | 5.95 | 1.68 | 6.52 | 1.24 | 0.623 | 0.009 |
| 13 | Inferior frontal gyrus, triangular part | Left | -0.94 | 6.79 | 0.47 | 8.41 | 0.87 | 0.634 | 0.004 |  | -0.44 | 6.72 | -0.16 | 7.06 | 0.52 | 0.799 | 0.004 |
| 14 | Inferior frontal gyrus, triangular part | Right | 0.36 | 6.43 | 0.36 | 7.35 | 0.05 | 0.963 | 0.000 |  | 1.53 | 6.63 | 2.09 | 7.32 | 0.22 | 0.832 | 0.002 |
| 15 | Inferior frontal gyrus, orbital part | Left | -2.02 | 5.90 | 1.74 | 6.82 | 11.70 | **0.014 *** | 0.052 |  | -1.71 | 5.76 | 0.19 | 6.57 | 0.66 | 0.755 | 0.005 |
| 16 | Inferior frontal gyrus, orbital part | Right | 0.01 | 7.09 | 1.69 | 7.04 | 0.92 | 0.626 | 0.004 |  | 0.78 | 6.93 | 2.19 | 7.84 | 0.29 | 0.832 | 0.002 |
| 17 | Rolandic operculum | Left | -0.19 | 6.24 | -0.67 | 6.03 | 0.60 | 0.692 | 0.003 |  | -0.39 | 5.61 | 1.20 | 6.54 | 1.74 | 0.622 | 0.013 |
| 18 | Rolandic operculum | Right | 0.19 | 5.18 | -0.22 | 6.07 | 0.00 | 0.989 | 0.000 |  | 1.05 | 5.37 | 1.15 | 5.20 | 0.10 | 0.841 | 0.001 |
| 19 | Supplementary motor area | Left | -0.18 | 5.09 | 0.61 | 6.43 | 0.77 | 0.661 | 0.004 |  | 0.41 | 5.24 | 1.26 | 5.51 | 1.78 | 0.622 | 0.014 |
| 20 | Supplementary motor area | Right | 0.34 | 6.77 | -0.15 | 7.35 | 0.01 | 0.989 | 0.000 |  | 1.15 | 6.92 | 0.89 | 6.47 | 0.24 | 0.832 | 0.002 |
| 21 | Olfactory cortex | Left | -1.77 | 5.86 | 1.00 | 6.60 | 3.85 | 0.153 | 0.018 |  | -1.39 | 5.44 | 0.96 | 6.23 | 2.44 | 0.545 | 0.019 |
| 22 | Olfactory cortex | Right | -0.71 | 6.06 | 0.30 | 6.12 | 0.27 | 0.840 | 0.001 |  | -0.83 | 6.22 | 0.55 | 5.96 | 0.41 | 0.832 | 0.003 |
| 23 | Superior frontal gyrus, medial | Left | 0.56 | 5.20 | 1.28 | 6.21 | 0.13 | 0.920 | 0.001 |  | 0.92 | 5.05 | 1.83 | 6.01 | 0.37 | 0.832 | 0.003 |
| 24 | Superior frontal gyrus, medial | Right | 0.10 | 5.17 | 0.48 | 6.14 | 0.07 | 0.961 | 0.000 |  | 0.72 | 5.10 | 0.74 | 5.59 | 0.19 | 0.832 | 0.001 |
| 25 | Superior frontal gyrus, medial orbital | Left | 1.08 | 5.45 | 1.39 | 4.78 | 0.06 | 0.961 | 0.000 |  | 1.51 | 4.75 | 1.76 | 4.98 | 0.09 | 0.841 | 0.001 |
| 26 | Superior frontal gyrus, medial orbital | Right | 1.42 | 4.70 | 1.11 | 4.89 | 1.14 | 0.572 | 0.005 |  | 1.24 | 4.57 | 0.57 | 4.22 | 1.06 | 0.639 | 0.008 |
| 27 | Gyrus rectus | Left | -0.79 | 4.24 | -0.19 | 3.96 | 0.19 | 0.883 | 0.001 |  | -0.26 | 3.98 | -0.39 | 3.79 | 0.74 | 0.740 | 0.006 |
| 28 | Gyrus rectus | Right | -0.83 | 4.22 | -0.23 | 4.02 | 0.77 | 0.661 | 0.004 |  | -0.32 | 4.17 | -0.65 | 4.09 | 0.67 | 0.755 | 0.005 |
| 29 | Insula | Left | -0.28 | 6.25 | 2.11 | 8.54 | 6.50 | 0.058 | 0.029 |  | -1.16 | 6.33 | 1.45 | 7.48 | 2.74 | 0.500 | 0.021 |
| 30 | Insula | Right | -0.52 | 7.80 | 4.94 | 8.16 | 19.26 | **0.002 *** | 0.083 |  | 0.14 | 8.36 | 3.28 | 8.68 | 2.45 | 0.545 | 0.019 |
| 31 | Anterior cingulate and paracingulate gyri | Left | 1.07 | 7.34 | 1.90 | 8.28 | 0.19 | 0.883 | 0.001 |  | 1.16 | 6.94 | 1.81 | 7.67 | 0.19 | 0.832 | 0.001 |
| 32 | Anterior cingulate and paracingulate gyri | Right | 0.62 | 7.13 | 1.43 | 8.56 | 0.04 | 0.973 | 0.000 |  | 2.02 | 6.76 | 1.04 | 7.26 | 1.13 | 0.623 | 0.009 |
| 33 | Median cingulate and paracingulate gyri | Left | 0.78 | 6.23 | 0.59 | 7.09 | 0.14 | 0.920 | 0.001 |  | 1.79 | 5.88 | 2.21 | 6.33 | 0.42 | 0.832 | 0.003 |
| 34 | Median cingulate and paracingulate gyri | Right | 0.92 | 6.82 | -0.20 | 7.50 | 0.49 | 0.715 | 0.002 |  | 1.69 | 6.57 | 1.49 | 6.65 | 0.18 | 0.832 | 0.001 |
| 35 | Posterior cingulate gyrus | Left | -0.33 | 5.72 | 1.19 | 6.47 | 4.02 | 0.150 | 0.018 |  | 0.18 | 5.25 | 1.19 | 5.76 | 1.61 | 0.622 | 0.012 |
| 36 | Posterior cingulate gyrus | Right | 1.03 | 6.04 | 3.18 | 5.94 | 7.32 | **0.048 *** | 0.033 |  | 2.07 | 5.88 | 2.74 | 5.92 | 0.78 | 0.739 | 0.006 |
| 37 | Hippocampus | Left | -0.82 | 4.08 | 0.15 | 5.07 | 7.01 | **0.049 *** | 0.032 |  | 0.36 | 3.76 | 0.15 | 4.45 | 0.82 | 0.734 | 0.006 |
| 38 | Hippocampus | Right | -0.25 | 6.34 | 0.46 | 6.66 | 0.00 | 0.995 | 0.000 |  | -0.53 | 6.18 | 0.94 | 6.61 | 0.41 | 0.832 | 0.003 |
| 39 | Parahippocampal gyrus | Left | -0.52 | 4.53 | 1.88 | 4.98 | 7.25 | **0.048 *** | 0.033 |  | -0.54 | 4.43 | 0.44 | 5.06 | 0.36 | 0.832 | 0.003 |
| 40 | Parahippocampal gyrus | Right | 0.68 | 5.26 | 2.76 | 5.83 | 3.91 | 0.153 | 0.018 |  | 0.85 | 5.83 | 0.87 | 5.96 | 0.32 | 0.832 | 0.002 |
| 41 | Amygdala | Left | -0.84 | 6.47 | 0.21 | 7.13 | 0.58 | 0.692 | 0.003 |  | 0.18 | 5.80 | -0.27 | 8.01 | 0.05 | 0.871 | 0.000 |
| 42 | Amygdala | Right | -0.23 | 4.62 | 0.10 | 5.48 | 0.00 | 0.989 | 0.000 |  | 0.16 | 4.29 | 1.22 | 5.12 | 1.44 | 0.623 | 0.011 |
| 43 | Calcarine fissure and surrounding cortex | Left | -0.10 | 5.45 | -0.21 | 7.12 | 0.05 | 0.963 | 0.000 |  | 0.44 | 5.51 | 0.41 | 5.77 | 0.09 | 0.841 | 0.001 |
| 44 | Calcarine fissure and surrounding cortex | Right | -0.18 | 5.38 | -0.01 | 6.00 | 0.55 | 0.692 | 0.003 |  | 0.59 | 4.81 | 0.41 | 5.52 | 0.04 | 0.875 | 0.000 |
| 45 | Cuneus | Left | -1.19 | 6.64 | 1.60 | 7.40 | 8.68 | **0.046 *** | 0.039 |  | -0.25 | 6.98 | 2.55 | 6.19 | 4.90 | 0.361 | 0.037 |
| 46 | Cuneus | Right | -1.29 | 6.91 | 1.61 | 7.41 | 11.65 | **0.014 *** | 0.052 |  | -0.10 | 7.25 | 1.80 | 6.37 | 3.75 | 0.387 | 0.028 |
| 47 | Lingual gyrus | Left | -0.06 | 4.13 | 0.25 | 4.97 | 0.54 | 0.692 | 0.003 |  | 0.31 | 3.93 | 0.71 | 4.01 | 0.29 | 0.832 | 0.002 |
| 48 | Lingual gyrus | Right | 0.00 | 3.74 | -0.27 | 4.76 | 0.03 | 0.976 | 0.000 |  | 0.41 | 3.61 | 0.41 | 4.05 | 0.15 | 0.841 | 0.001 |
| 49 | Superior occipital gyrus | Left | -0.94 | 5.86 | -0.01 | 6.70 | 0.91 | 0.626 | 0.004 |  | -0.64 | 6.04 | 2.49 | 6.14 | 7.69 | 0.287 | 0.056 |
| 50 | Superior occipital gyrus | Right | -1.33 | 6.09 | 0.85 | 6.92 | 7.18 | **0.048 *** | 0.032 |  | -0.70 | 6.03 | 1.65 | 5.94 | 6.66 | 0.329 | 0.049 |
| 51 | Middle occipital gyrus | Left | -0.82 | 6.82 | 0.27 | 7.78 | 2.08 | 0.357 | 0.010 |  | -0.63 | 6.75 | 1.78 | 6.54 | 4.69 | 0.361 | 0.035 |
| 52 | Middle occipital gyrus | Right | -0.87 | 7.90 | 0.68 | 9.39 | 4.01 | 0.150 | 0.018 |  | -0.22 | 7.91 | 1.68 | 7.74 | 1.61 | 0.622 | 0.012 |
| 53 | Inferior occipital gyrus | Left | 0.19 | 5.70 | -0.73 | 6.74 | 0.28 | 0.840 | 0.001 |  | 0.52 | 5.43 | 1.00 | 5.62 | 0.05 | 0.871 | 0.000 |
| 54 | Inferior occipital gyrus | Right | 0.68 | 4.14 | 0.48 | 5.22 | 0.00 | 0.989 | 0.000 |  | 0.77 | 4.20 | 1.08 | 4.51 | 0.21 | 0.832 | 0.002 |
| 55 | Fusiform gyrus | Left | 0.05 | 4.51 | -1.12 | 5.12 | 2.33 | 0.313 | 0.011 |  | 0.19 | 4.31 | -0.11 | 4.75 | 0.03 | 0.888 | 0.000 |
| 56 | Fusiform gyrus | Right | 0.10 | 5.01 | -0.49 | 6.00 | 0.01 | 0.989 | 0.000 |  | 0.25 | 5.02 | 0.81 | 4.44 | 1.18 | 0.623 | 0.009 |
| 57 | Postcentral gyrus | Left | 0.16 | 6.81 | 0.29 | 7.45 | 0.01 | 0.989 | 0.000 |  | 0.93 | 7.01 | 2.49 | 7.26 | 1.24 | 0.623 | 0.010 |
| 58 | Postcentral gyrus | Right | -0.01 | 5.53 | 0.54 | 6.29 | 1.02 | 0.599 | 0.005 |  | 0.55 | 5.49 | 2.62 | 5.75 | 5.22 | 0.359 | 0.039 |
| 59 | Superior parietal gyrus | Left | -0.93 | 5.87 | 0.53 | 6.53 | 2.36 | 0.313 | 0.011 |  | -0.65 | 5.81 | 1.72 | 5.75 | 4.27 | 0.387 | 0.032 |
| 60 | Superior parietal gyrus | Right | -0.07 | 4.78 | 2.10 | 5.55 | 7.95 | **0.047 *** | 0.036 |  | 0.48 | 4.80 | 2.32 | 4.82 | 3.76 | 0.387 | 0.028 |
| 61 | Inferior parietal, but supramarginal and angular gyri | Left | 0.79 | 7.86 | 1.99 | 9.42 | 1.17 | 0.572 | 0.005 |  | 1.85 | 8.47 | 2.76 | 7.91 | 0.20 | 0.832 | 0.002 |
| 62 | Inferior parietal, but supramarginal and angular gyri | Right | 0.67 | 6.67 | 2.28 | 8.05 | 2.74 | 0.271 | 0.013 |  | 1.25 | 6.35 | 3.29 | 5.59 | 2.12 | 0.600 | 0.016 |
| 63 | Supramarginal gyrus | Left | 1.28 | 8.31 | -0.04 | 8.25 | 1.43 | 0.500 | 0.007 |  | 1.61 | 8.46 | 1.57 | 7.90 | 0.03 | 0.888 | 0.000 |
| 64 | Supramarginal gyrus | Right | -0.24 | 6.32 | 0.92 | 7.86 | 2.02 | 0.362 | 0.009 |  | 0.04 | 6.37 | 1.89 | 5.93 | 2.06 | 0.600 | 0.016 |
| 65 | Angular gyrus | Left | -0.44 | 5.28 | 1.27 | 5.50 | 4.26 | 0.139 | 0.020 |  | 0.00 | 5.45 | 1.67 | 5.01 | 1.87 | 0.622 | 0.014 |
| 66 | Angular gyrus | Right | -0.16 | 5.49 | 1.37 | 6.05 | 5.14 | 0.089 | 0.023 |  | 0.32 | 5.38 | 1.27 | 5.24 | 1.54 | 0.623 | 0.012 |
| 67 | Precuneus | Left | -0.30 | 5.68 | 1.62 | 6.90 | 8.03 | **0.047 *** | 0.036 |  | 0.23 | 5.80 | 2.89 | 6.01 | 8.17 | 0.287 | 0.060 |
| 68 | Precuneus | Right | -0.77 | 6.92 | 1.02 | 8.75 | 5.31 | 0.089 | 0.024 |  | 0.10 | 6.67 | 1.87 | 7.07 | 1.75 | 0.622 | 0.013 |
| 69 | Paracentral lobule | Left | 0.24 | 3.91 | -0.58 | 4.41 | 1.67 | 0.435 | 0.008 |  | -0.15 | 3.90 | -0.02 | 3.74 | 0.21 | 0.832 | 0.002 |
| 70 | Paracentral lobule | Right | 0.29 | 1.56 | 0.05 | 1.66 | 0.71 | 0.669 | 0.003 |  | 0.48 | 1.56 | 0.35 | 1.49 | 0.01 | 0.947 | 0.000 |
| 71 | Caudate nucleus | Left | -2.12 | 6.96 | 0.41 | 6.78 | 1.90 | 0.383 | 0.009 |  | -1.63 | 7.32 | 1.93 | 6.21 | 5.41 | 0.359 | 0.040 |
| 72 | Caudate nucleus | Right | -2.20 | 6.75 | 0.89 | 6.62 | 7.47 | **0.048 *** | 0.034 |  | -1.74 | 6.84 | 0.93 | 6.54 | 3.64 | 0.387 | 0.027 |
| 73 | Lenticular nucleus putamen | Left | -0.09 | 5.21 | -1.21 | 5.38 | 2.62 | 0.283 | 0.012 |  | 0.39 | 5.18 | 1.00 | 5.38 | 0.23 | 0.832 | 0.002 |
| 74 | Lenticular nucleus putamen | Right | -0.30 | 6.05 | -0.63 | 6.31 | 0.07 | 0.961 | 0.000 |  | 0.94 | 5.87 | 0.28 | 6.52 | 0.73 | 0.740 | 0.006 |
| 75 | Lenticular nucleus, pallidum | Left | -0.34 | 5.00 | -1.78 | 5.36 | 5.17 | 0.089 | 0.024 |  | 0.24 | 5.17 | 0.60 | 4.67 | 0.12 | 0.841 | 0.001 |
| 76 | Lenticular nucleus, pallidum | Right | -0.19 | 4.94 | -0.52 | 5.74 | 0.11 | 0.931 | 0.000 |  | 0.16 | 5.14 | 0.27 | 5.05 | 0.09 | 0.841 | 0.001 |
| 77 | Thalamus | Left | -0.66 | 6.92 | -0.44 | 6.62 | 0.00 | 0.989 | 0.000 |  | 0.15 | 6.92 | 1.19 | 7.06 | 1.20 | 0.623 | 0.009 |
| 78 | Thalamus | Right | -0.21 | 6.51 | 1.35 | 6.48 | 2.55 | 0.288 | 0.012 |  | -0.32 | 6.92 | 2.24 | 6.34 | 6.13 | 0.329 | 0.045 |
| 79 | Heschl gyrus | Left | -0.63 | 4.18 | 0.93 | 4.53 | 8.95 | **0.046 *** | 0.040 |  | -0.72 | 3.99 | 0.37 | 4.31 | 1.69 | 0.622 | 0.013 |
| 80 | Heschl gyrus | Right | -1.15 | 6.50 | 2.30 | 7.38 | 12.94 | **0.014 *** | 0.057 |  | -1.20 | 6.56 | 1.75 | 7.46 | 3.60 | 0.387 | 0.027 |
| 81 | Superior temporal gyrus | Left | -0.99 | 6.90 | 1.25 | 8.44 | 2.75 | 0.271 | 0.013 |  | -0.88 | 6.45 | 1.61 | 7.07 | 3.38 | 0.410 | 0.026 |
| 82 | Superior temporal gyrus | Right | -0.33 | 7.09 | 3.57 | 9.41 | 12.33 | **0.014 *** | 0.054 |  | -0.29 | 6.73 | 2.09 | 7.92 | 3.67 | 0.387 | 0.028 |
| 83 | Temporal pole: superior temporal gyrus | Left | -1.09 | 5.92 | 1.37 | 5.42 | 5.78 | 0.076 | 0.026 |  | -0.60 | 5.30 | -0.01 | 6.09 | 0.06 | 0.871 | 0.000 |
| 84 | Temporal pole: superior temporal gyrus | Right | -1.09 | 7.12 | 1.61 | 7.59 | 6.01 | 0.071 | 0.027 |  | -0.34 | 7.07 | 0.50 | 7.38 | 0.31 | 0.832 | 0.002 |
| 85 | Middle temporal gyrus | Left | -0.93 | 9.13 | -0.94 | 11.53 | 0.00 | 0.989 | 0.000 |  | -0.35 | 9.18 | 1.26 | 8.71 | 0.57 | 0.782 | 0.004 |
| 86 | Middle temporal gyrus | Right | 0.45 | 6.89 | 1.53 | 8.07 | 2.74 | 0.271 | 0.013 |  | 1.22 | 6.47 | 0.76 | 6.79 | 0.00 | 0.991 | 0.000 |
| 87 | Temporal pole: middle temporal gyrus | Left | -2.29 | 5.33 | -0.48 | 6.15 | 6.88 | **0.049 *** | 0.031 |  | -0.55 | 5.37 | -1.63 | 5.28 | 1.40 | 0.623 | 0.011 |
| 88 | Temporal pole: middle temporal gyrus | Right | 0.61 | 5.63 | -1.75 | 5.13 | 8.03 | **0.047 *** | 0.036 |  | 0.36 | 5.20 | -0.78 | 5.22 | 2.09 | 0.600 | 0.016 |
| 89 | Inferior temporal gyrus | Left | -0.80 | 7.99 | 2.60 | 7.65 | 7.40 | **0.048 *** | 0.033 |  | -0.65 | 6.93 | 0.99 | 7.02 | 0.38 | 0.832 | 0.003 |
| 90 | Inferior temporal gyrus | Right | -0.52 | 7.14 | 1.91 | 8.11 | 5.71 | 0.076 | 0.026 |  | -0.44 | 7.34 | 1.65 | 5.68 | 1.13 | 0.623 | 0.009 |

Abbreviations: AAL, automated anatomical labeling; BD, bipolar disorder; FDR, false discovery rate; MDD, major depressive disorder; SD, standard deviation.

Significant differences after FDR correction are indicated with * (adjusted *p* < 0.05).

**Supplementary Table 6. Brain age gap differences between individuals with BD and MDD compared to age- and sex-matched healthy controls in 48 models for fractional anisotropy map.**

| **JHU-ICBM-Label number** | **White matter tracts** | **Left/Right** | **Healthy controls**  **(*n* = 110)** | | **BD group**  **(*n* = 110)** | | ***F*** | **adjusted *p*** | ***partial η^2^*** |  | **Healthy controls**  **(*n* = 68)** | | **MDD group**  **(*n* = 68)** | | ***F*** | **adjusted *p*** | ***partial η^2^*** |
| --- | --- | --- | --- | --- | --- | --- | --- | --- | --- | --- | --- | --- | --- | --- | --- | --- | --- |
|  |  |  | **Mean (years)** | **SD** | **Mean (years)** | **SD** |  |  |  |  | **Mean (years)** | **SD** | **Mean (years)** | **SD** |  |  |  |
| 1 | Middle cerebellar peduncle | – | -2.92 | 8.11 | 11.24 | 6.60 | 171.27 | **< 0.001 *** | 0.445 |  | -4.80 | 8.33 | 11.36 | 6.94 | 131.82 | **< 0.001 *** | 0.503 |
| 2 | Pontine crossing tract | – | -2.47 | 5.07 | 0.37 | 7.02 | 4.93 | **0.031 *** | 0.022 |  | -2.60 | 4.00 | 0.64 | 7.03 | 7.37 | **0.011 *** | 0.054 |
| 3 | Genu of corpus callosum | – | -3.51 | 7.36 | 4.62 | 9.58 | 29.38 | **< 0.001 *** | 0.121 |  | -3.67 | 6.27 | 1.84 | 9.61 | 13.06 | **0.001 *** | 0.091 |
| 4 | Body of corpus callosum | – | -3.64 | 7.07 | 4.70 | 9.68 | 37.36 | **< 0.001 *** | 0.149 |  | -4.03 | 6.70 | 2.04 | 8.39 | 20.18 | **< 0.001 *** | 0.134 |
| 5 | Splenium of corpus callosum | – | -2.97 | 7.83 | 4.86 | 8.61 | 28.82 | **< 0.001 *** | 0.119 |  | -3.77 | 7.13 | 2.37 | 8.30 | 14.44 | **0.001 *** | 0.100 |
| 6 | Fornix (fornix column and body of fornix) | – | -2.84 | 8.28 | 4.73 | 8.53 | 31.59 | **< 0.001 *** | 0.129 |  | -3.16 | 8.47 | 3.47 | 9.09 | 13.16 | **0.001 *** | 0.092 |
| 7 | Corticospinal tract | Right | -2.56 | 8.78 | 11.90 | 6.60 | 169.89 | **< 0.001 *** | 0.443 |  | -4.62 | 9.00 | 12.25 | 5.78 | 140.16 | **< 0.001 *** | 0.519 |
| 8 | Corticospinal tract | Left | -1.97 | 8.31 | 3.24 | 6.74 | 23.10 | **< 0.001 *** | 0.097 |  | -3.68 | 8.80 | 2.21 | 7.01 | 15.40 | **0.001 *** | 0.106 |
| 9 | Medial lemniscus | Right | -1.06 | 8.13 | 2.14 | 8.16 | 5.18 | **0.027 *** | 0.024 |  | -0.45 | 8.33 | -1.31 | 8.09 | 0.28 | 0.636 | 0.002 |
| 10 | Medial lemniscus | Left | -1.84 | 8.76 | 2.52 | 9.02 | 8.15 | **0.006 *** | 0.037 |  | -1.66 | 9.10 | -1.30 | 7.88 | 0.07 | 0.785 | 0.001 |
| 11 | Inferior cerebellar peduncle | Right | -2.71 | 7.95 | 3.26 | 7.04 | 33.87 | **< 0.001 *** | 0.137 |  | -4.74 | 7.94 | 0.83 | 7.38 | 15.43 | **0.001 *** | 0.106 |
| 12 | Inferior cerebellar peduncle | Left | -5.34 | 9.13 | -1.62 | 6.84 | 10.16 | **0.002 *** | 0.045 |  | -3.34 | 9.62 | -2.26 | 7.36 | 0.24 | 0.652 | 0.002 |
| 13 | Superior cerebellar peduncle | Right | -0.30 | 9.32 | 0.66 | 10.20 | 0.00 | 0.944 | 0.000 |  | -0.79 | 9.90 | -2.81 | 8.58 | 1.57 | 0.249 | 0.012 |
| 14 | Superior cerebellar peduncle | Left | -1.61 | 10.38 | 0.33 | 11.11 | 0.43 | 0.522 | 0.002 |  | -1.49 | 11.07 | -3.20 | 10.07 | 0.88 | 0.383 | 0.007 |
| 15 | Cerebral peduncle | Right | -1.23 | 8.19 | 4.03 | 10.23 | 9.17 | **0.004 *** | 0.041 |  | -1.49 | 8.61 | 0.56 | 9.92 | 1.27 | 0.292 | 0.010 |
| 16 | Cerebral peduncle | Left | -1.33 | 7.74 | 3.19 | 8.63 | 8.07 | **0.006 *** | 0.036 |  | -1.47 | 7.72 | 1.76 | 8.09 | 4.99 | **0.035 *** | 0.037 |
| 17 | Anterior limb of internal capsule | Right | -2.87 | 8.41 | 4.18 | 10.37 | 17.07 | **< 0.001 *** | 0.074 |  | -3.06 | 7.62 | 2.65 | 9.90 | 8.48 | **0.008 *** | 0.061 |
| 18 | Anterior limb of internal capsule | Left | -0.73 | 7.25 | 4.35 | 8.84 | 6.68 | **0.013 *** | 0.030 |  | -1.33 | 6.49 | 3.36 | 9.69 | 5.35 | **0.030 *** | 0.040 |
| 19 | Posterior limb of internal capsule | Right | -1.90 | 7.50 | 3.10 | 9.08 | 13.64 | **< 0.001 *** | 0.060 |  | -2.64 | 6.54 | 0.25 | 8.47 | 5.95 | **0.022 *** | 0.044 |
| 20 | Posterior limb of internal capsule | Left | -1.95 | 7.80 | 2.49 | 8.62 | 12.73 | **0.001 *** | 0.056 |  | -2.72 | 7.80 | 0.93 | 8.79 | 5.92 | **0.022 *** | 0.044 |
| 21 | Retrolenticular part of internal capsule | Right | -3.35 | 8.41 | 1.82 | 9.06 | 9.52 | **0.003 *** | 0.043 |  | -4.18 | 8.47 | 2.05 | 8.24 | 13.64 | **0.001 *** | 0.095 |
| 22 | Retrolenticular part of internal capsule | Left | -2.75 | 8.93 | 2.12 | 8.27 | 11.56 | **0.001 *** | 0.051 |  | -4.53 | 8.51 | 3.15 | 8.85 | 19.91 | **< 0.001 *** | 0.133 |
| 23 | Anterior corona radiata | Right | -3.36 | 7.73 | 4.55 | 9.76 | 26.09 | **< 0.001 *** | 0.109 |  | -3.59 | 7.33 | 0.75 | 8.96 | 8.42 | **0.008 *** | 0.061 |
| 24 | Anterior corona radiata | Left | -2.75 | 6.90 | 3.39 | 9.27 | 16.11 | **< 0.001 *** | 0.070 |  | -3.34 | 6.57 | 1.01 | 8.42 | 8.30 | **0.008 *** | 0.060 |
| 25 | Superior corona radiata | Right | -1.98 | 6.91 | 3.66 | 9.17 | 14.36 | **< 0.001 *** | 0.063 |  | -2.25 | 6.32 | 1.30 | 9.33 | 7.65 | **0.010 *** | 0.056 |
| 26 | Superior corona radiata | Left | -2.76 | 7.28 | 3.64 | 9.41 | 22.87 | **< 0.001 *** | 0.097 |  | -3.46 | 7.32 | 1.76 | 8.80 | 16.00 | **0.001 *** | 0.110 |
| 27 | Posterior corona radiata | Right | -2.69 | 7.31 | 4.32 | 8.65 | 26.36 | **< 0.001 *** | 0.110 |  | -3.66 | 6.90 | 1.49 | 7.86 | 13.29 | **0.001 *** | 0.093 |
| 28 | Posterior corona radiata | Left | -2.92 | 6.71 | 4.22 | 8.34 | 30.36 | **< 0.001 *** | 0.124 |  | -3.81 | 6.63 | 1.38 | 7.80 | 18.38 | **< 0.001 *** | 0.124 |
| 29 | Posterior thalamic radiation | Right | -1.26 | 7.53 | 4.85 | 6.05 | 29.99 | **< 0.001 *** | 0.123 |  | -2.12 | 6.88 | 2.77 | 6.95 | 15.20 | **0.001 *** | 0.105 |
| 30 | Posterior thalamic radiation | Left | -1.84 | 7.30 | 4.31 | 8.89 | 25.00 | **< 0.001 *** | 0.105 |  | -2.76 | 7.24 | 3.31 | 9.02 | 18.62 | **< 0.001 *** | 0.125 |
| 31 | Sagittal stratum | Right | -1.61 | 7.38 | 4.49 | 9.38 | 16.95 | **< 0.001 *** | 0.073 |  | -1.68 | 7.41 | 3.26 | 8.39 | 12.34 | **0.001 *** | 0.087 |
| 32 | Sagittal stratum | Left | -2.48 | 8.03 | 4.88 | 9.79 | 23.89 | **< 0.001 *** | 0.100 |  | -2.92 | 7.49 | 3.10 | 9.99 | 9.87 | **0.004 *** | 0.071 |
| 33 | External capsule | Right | -1.88 | 7.99 | 4.50 | 9.91 | 16.94 | **< 0.001 *** | 0.073 |  | -2.93 | 7.99 | 4.07 | 9.74 | 16.28 | **0.001 *** | 0.111 |
| 34 | External capsule | Left | -1.93 | 7.20 | 4.51 | 9.34 | 21.45 | **< 0.001 *** | 0.091 |  | -2.11 | 7.05 | 2.86 | 9.29 | 11.15 | **0.002 *** | 0.079 |
| 35 | Cingulum (cingulate gyrus) | Right | -1.16 | 7.43 | 3.13 | 9.03 | 6.13 | **0.016 *** | 0.028 |  | -1.70 | 7.43 | 1.95 | 8.77 | 3.92 | 0.060 | 0.029 |
| 36 | Cingulum (cingulate gyrus) | Left | -0.96 | 6.74 | 3.37 | 8.70 | 8.14 | **0.006 *** | 0.037 |  | -2.71 | 6.45 | 0.80 | 7.87 | 4.95 | **0.035 *** | 0.037 |
| 37 | Cingulum (hippocampus) | Right | -0.56 | 8.47 | 2.71 | 8.74 | 3.84 | 0.056 | 0.018 |  | -1.21 | 8.58 | 0.25 | 7.46 | 0.17 | 0.696 | 0.001 |
| 38 | Cingulum (hippocampus) | Left | -0.51 | 8.53 | 3.94 | 8.09 | 9.49 | **0.003 *** | 0.042 |  | -1.97 | 8.73 | 0.55 | 7.32 | 1.43 | 0.268 | 0.011 |
| 39 | Fornix (cres/stria terminalis) | Right | -3.09 | 7.83 | 5.13 | 10.20 | 36.71 | **< 0.001 *** | 0.146 |  | -4.65 | 6.91 | 0.66 | 10.23 | 13.42 | **0.001 *** | 0.094 |
| 40 | Fornix (cres/stria terminalis) | Left | -1.87 | 8.67 | 5.09 | 9.70 | 22.23 | **< 0.001 *** | 0.094 |  | -3.33 | 7.50 | 2.03 | 9.66 | 11.42 | **0.002 *** | 0.081 |
| 41 | Superior longitudinal fasciculus | Right | -3.26 | 6.95 | 1.58 | 8.57 | 14.27 | **< 0.001 *** | 0.063 |  | -4.06 | 7.12 | 0.80 | 9.26 | 8.22 | **0.008 *** | 0.059 |
| 42 | Superior longitudinal fasciculus | Left | -2.27 | 8.21 | 1.84 | 8.20 | 14.00 | **< 0.001 *** | 0.061 |  | -3.22 | 7.32 | 0.45 | 8.64 | 6.85 | **0.014 *** | 0.050 |
| 43 | Superior fronto-occipital fasciculus | Right | -1.72 | 7.33 | 4.39 | 9.61 | 16.08 | **< 0.001 *** | 0.070 |  | -2.30 | 7.13 | 3.25 | 9.96 | 8.46 | **0.008 *** | 0.061 |
| 44 | Superior fronto-occipital fasciculus | Left | -2.15 | 7.55 | 1.51 | 8.08 | 6.63 | **0.013 *** | 0.030 |  | -2.95 | 7.17 | 0.85 | 10.61 | 4.46 | **0.045 *** | 0.033 |
| 45 | Uncinate fasciculus | Right | -2.25 | 7.15 | 1.23 | 8.98 | 2.42 | 0.127 | 0.011 |  | -2.71 | 6.98 | 2.45 | 10.79 | 8.35 | **0.008 *** | 0.060 |
| 46 | Uncinate fasciculus | Left | -0.84 | 6.26 | 1.89 | 7.54 | 2.72 | 0.108 | 0.013 |  | -0.56 | 6.62 | 3.99 | 7.54 | 11.05 | **0.002 *** | 0.078 |
| 47 | Tapetum | Right | -3.34 | 6.72 | 4.70 | 8.03 | 46.48 | **< 0.001 *** | 0.178 |  | -4.47 | 6.33 | 1.59 | 7.27 | 24.09 | **< 0.001 *** | 0.156 |
| 48 | Tapetum | Left | -3.22 | 7.20 | 3.00 | 8.64 | 29.69 | **< 0.001 *** | 0.122 |  | -4.24 | 7.29 | 0.76 | 8.04 | 13.34 | **0.001 *** | 0.093 |

Abbreviations: BD, bipolar disorder; FDR, false discovery rate; MDD, major depressive disorder; SD, standard deviation.

Significant differences after FDR correction are indicated with * (adjusted *p* < 0.05).

**References**

Hua, K., Zhang, J., Wakana, S., Jiang, H., Li, X., Reich, D. S., ... Mori, S. (2008). Tract probability maps in stereotaxic spaces: Analyses of white matter anatomy and tract-specific quantification. NeuroImage, 39(1), 336–347. https://doi.org/10.1016/j.neuroimage.2007.07.053

Jenkinson, M., Beckmann, C. F., Behrens, T. E., Woolrich, M. W., & Smith, S. M. (2012). FSL. NeuroImage, 62(2), 782–790. https://doi.org/10.1016/j.neuroimage.2011.09.015

Shen, C.-L., Tsai, S.-J., Lin, C.-P., & Yang, A. C. (2023). Progressive brain abnormalities in schizophrenia across different illness periods: A structural and functional MRI study. Schizophrenia, 9(1), 2. https://doi.org/10.1038/s41537-022-00301-2

Tzourio-Mazoyer, N., Landeau, B., Papathanassiou, D., Crivello, F., Etard, O., Delcroix, N., ... Joliot, M. (2002). Automated anatomical labeling of activations in SPM using a macroscopic anatomical parcellation of the MNI MRI single-subject brain. NeuroImage, 15(1), 273–289. https://doi.org/10.1006/nimg.2001.0978

Wakana, S., Caprihan, A., Panzenboeck, M. M., Fallon, J. H., Perry, M., Gollub, R. L., ... Dubey, P. (2007). Reproducibility of quantitative tractography methods applied to cerebral white matter. NeuroImage, 36(3), 630–644. https://doi.org/10.1016/j.neuroimage.2007.02.049

Yan, C.-G., Wang, X.-D., Zuo, X.-N., & Zang, Y.-F. (2016). DPABI: Data processing & analysis for (resting-state) brain imaging. Neuroinformatics, 14, 339–351. https://doi.org/10.1007/s12021-016-9299-4

Yang, A. C., Hong, C. J., Liou, Y. J., Huang, K. L., Huang, C. C., Liu, M. E., ... Lin, C. P. (2015). Decreased resting-state brain activity complexity in schizophrenia characterized by both increased regularity and randomness. Human Brain Mapping, 36(6), 2174–2186. https://doi.org/10.1002/hbm.22763

Yang, A. C., Tsai, S.-J., Lin, C.-P., Peng, C.-K., & Huang, N. E. (2018). Frequency and amplitude modulation of resting-state fMRI signals and their functional relevance in normal aging. Neurobiology of Aging, 70, 59–69. https://doi.org/10.1016/j.neurobiolaging.2018.05.017

Zhu, J.-D., Huang, C.-W., Chang, H.-I., Tsai, S.-J., Huang, S.-H., Hsu, S.-W., ... Yang, A. C. (2022). Functional MRI and ApoE4 genotype for predicting cognitive decline in amyloid-positive individuals. Therapeutic Advances in Neurological Disorders, 15, 17562864221138154. https://doi.org/10.1177/17562864221138154

Zhu, J.-D., Tsai, S.-J., Lin, C.-P., Lee, Y.-J., & Yang, A. C. (2023). Predicting aging trajectories of decline in brain volume, cortical thickness and fractional anisotropy in schizophrenia. Schizophrenia, 9(1), 1. https://doi.org/10.1038/s41537-022-00299-7

Zhu, J.-D., Wu, Y.-F., Tsai, S.-J., Lin, C.-P., & Yang, A. C. (2023). Investigating brain aging trajectory deviations in different brain regions of individuals with schizophrenia using multimodal magnetic resonance imaging and brain-age prediction: A multicenter study. Translational Psychiatry, 13(1), 82. https://doi.org/10.1038/s41398-023-02408-z
